# Genomic Characterization of the Fruity Aroma Gene, *FaFAD1*, Reveals a Gene Dosage Effect on γ-Decalactone Production in Strawberry (*Fragaria* × *ananassa*)

**DOI:** 10.3389/fpls.2021.639345

**Published:** 2021-05-04

**Authors:** Youngjae Oh, Christopher R. Barbey, Saket Chandra, Jinhe Bai, Zhen Fan, Anne Plotto, Jeremy Pillet, Kevin M. Folta, Vance M. Whitaker, Seonghee Lee

**Affiliations:** ^1^Department of Horticultural Sciences, Institute of Food and Agricultural Sciences (IFAS) Gulf Coast Research and Education Center, University of Florida, Wimauma, FL, United States; ^2^Department of Horticultural Sciences, University of Florida, Gainesville, FL, United States; ^3^Horticultural Research Laboratory, Agricultural Research Service (ARS), U.S. Department of Agriculture (USDA), Fort Pierce, FL, United States

**Keywords:** high-resolution melting marker, SNP array, GC-MASS, genome-wide association analysis, octoploid strawberry, fruit flavor

## Abstract

Strawberries produce numerous volatile compounds that contribute to the unique flavors of fruits. Among the many volatiles, γ-decalactone (γ-D) has the greatest contribution to the characteristic fruity aroma in strawberry fruit. The presence or absence of γ-D is controlled by a single locus, *FaFAD1*. However, this locus has not yet been systematically characterized in the octoploid strawberry genome. It has also been reported that the volatile content greatly varies among the strawberry varieties possessing *FaFAD1*, suggesting that another genetic factor could be responsible for the different levels of γ-D in fruit. In this study, we explored the genomic structure of *FaFAD1* and determined the allele dosage of *FaFAD1* that regulates variations of γ-D production in cultivated octoploid strawberry. The genome-wide association studies confirmed the major locus *FaFAD1* that regulates the γ-D production in cultivated strawberry. With the hybrid capture-based next-generation sequencing analysis, a major presence–absence variation of *FaFAD1* was discovered among γ-D producers and non-producers. To explore the genomic structure of *FaFAD1* in the octoploid strawberry, three bacterial artificial chromosome (BAC) libraries were developed. A deletion of 8,262 bp was consistently found in the *FaFAD1* region of γ-D non-producing varieties. With the newly developed InDel-based codominant marker genotyping, along with γ-D metabolite profiling data, we revealed the impact of gene dosage effect for the production of γ-D in the octoploid strawberry varieties. Altogether, this study provides systematic information of the prominent role of *FaFAD1* presence and absence polymorphism in producing γ-D and proposes that both alleles of *FaFAD1* are required to produce the highest content of fruity aroma in strawberry fruit.

## Introduction

Flavor and aroma are important characteristics of fruit quality in the cultivated octoploid strawberry (*Fragaria* × *ananassa*). Over 350 volatile compounds have been characterized in strawberry fruit including alcohols, aldehydes, esters, ketones, lactones, and terpenes (Latrasse, 1991; Urrutia et al., 2017). The major aroma compounds are identified as esters, lactones, furanones, sulfur compounds, and terpenoids (Perez et al., 1992; Zabetakis and Holden, 1997; Dong et al., 2013; Cruz-Rus et al., 2017). Among these flavor and aroma compounds, γ-decalactone (γ-D) has a desirable “fruity,” “sweet,” or “peach-like” aroma in strawberry fruit (Larsen et al., 1992; Jetti et al., 2007; Ulrich et al., 2007; Jouquand et al., 2008; Olbricht et al., 2008). Presence or absence of γ-D in strawberry fruit is controlled by a single gene, *FaFAD1*, encoding omega-6 fatty acid desaturase. This gene was mapped to the linkage group III (LG3) in the octoploid strawberry (Zorrilla-Fontanesi et al., 2012; Chambers et al., 2014; Sánchez-Sevilla et al., 2014). The abundance of γ-D is closely linked to *FaFAD1* transcript levels, and a PCR-based marker has been developed, which mostly predicts presence/absence of both *FAD1* and γ-D abundance (Cruz-Rus et al., 2017). However, the causal mutation that modulates γ-D abundance remains uncharacterized and prevents the development of a functional marker. While it has been hypothesized that the lack of γ-D is due to a deletion of the *FaFAD1* (Chambers et al., 2014; Sánchez-Sevilla et al., 2014), the precise genomic context of the deletion has not been characterized. Therefore, additional sequencing and comparative analyses are required to characterize the genomic structure of the *FaFAD1* region.

The concentration of γ-D varies widely among different γ-D-producing accessions (Chambers et al., 2014); however, the potential genetic and environmental causes for this variation have not been identified. Gene copy number variation (CNV) and gene dosage effects could be major sources of trait variation, with examples including muscat flavor in grapevine, aluminum tolerance in maize, and flowering time in wheat (Díaz et al., 2012; Maron et al., 2013; Emanuelli et al., 2014). In previous studies, several gene-specific sequence-tagged site (STS) and high-resolution melting (HRM) markers accurately predicted the presence and absence of *FaFAD1* in strawberry cultivars (Sánchez-Sevilla et al., 2014; Noh et al., 2017). However, these dominant markers could not explain any possible gene dosage effects for variation in γ-D content. A functional codominant marker could help characterize gene dosage effects and provide increased efficiency in marker-assisted breeding.

The modern cultivated strawberry (*Fragaria* × *ananassa*) is an allo-octoploid (2n = 8 × = 56), hybridization between a Chilean strawberry (*F. chiloensis*) and a North American native strawberry (*F. virginiana*) (Njunguna et al., 2013). The genome of diploid progenitor species *Fragaria vesca* was sequenced (Shulaev et al., 2011) and has been used as a diploid reference genome for gene-trait association studies in *F.* × *ananassa* and toward DNA marker development. However, the octoploid *F.* × *ananassa* genome is far more complicated than its diploid progenitors. In the last year, the chromosome-scale *F.* × *ananassa* ‘Camarosa’ reference genome was developed, and *F. vesca* was shown to be the dominant diploid progenitor in terms of gene content, expression abundance, and genetic control for metabolic and disease resistance traits (Edger et al., 2019). Two other diploid progenitors, *F. iinumae* and *F. nipponica*, have been recently fully sequenced^1^. The other new reference genome will serve as a powerful genetic resource to unravel complexity of the octoploid cultivated strawberry genome for gene-trait association studies.

Unlike the diploid genome, polyploid genomes have complex subgenomes that originated from the same/different diploid ancestors (Wendel, 1989, 2000; Levy and Feldman, 2002; Wendel and Cronn, 2003; Aharoni et al., 2004; Edger et al., 2019). Thus, polyploid subgenomes will likely show variation in copy numbers at homoeologous loci. Duplicated homologs are often observed in different subgenomes, which leads to difficulties in the isolation of subgenome-specific sequences or gene variations related to phenotypic trait variations. In particular, when certain genomic information is missing in a reference genome, bacterial artificial chromosome (BAC) libraries carrying large insert genomic DNA can be an effective tool for map-based gene cloning and characterization of target genomic regions in polyploids (Klymiuk et al., 2018; Liu et al., 2018). Because of the complexity of the octoploid strawberry genome, the recent construction of high-quality subgenome-specific reference sequences significantly facilitates identification of quantitative trait loci (QTL) and development of DNA markers tightly linked to agronomical important traits. The current octoploid reference genome (cv. Camarosa) is not phased genome assembly, and thus it does not contain complete genome information of each haplotype. There is another octoploid reference sequence available from Japanese cultivar ‘Reikou’, which is haplotype-resolved assembly^2^, but this draft genome sequence has not been fully characterized. Due to the high levels of genetic variations present in different breeding germplasm, it is not possible to explain all the genetic complexity of the octoploid cultivated strawberry with only two reference genomes. In addition, the two octoploid strawberry reference genomes do not have the *FaFAD1* region, and this hinders dissection of the functional sequence variations linked to γ-D content among cultivated strawberry varieties. Because of these limitations, chromosome specific BAC libraries can be especially valuable in polyploid varieties as they avoid the problem of homoeology (Wang et al., 2007), and they further identify genomic regions of target traits that are absent in the reference genome sequence (Gordon et al., 1999).

This report presents a comprehensive characterization of the genomic context of the *FaFAD1* deletion, along with functional data showing that the gene is both necessary and sufficient for γ-D production. A dosage effect has been observed between the allelic states, allowing the development of co-dominant DNA markers that can be used to assess a genotype’s ability to produce this important flavor volatile.

## Results

### Physical Mapping of the Major Locus *FaFAD1* That Regulates the Production of γ-D in the Octoploid Strawberry

**FIGURE 1:**
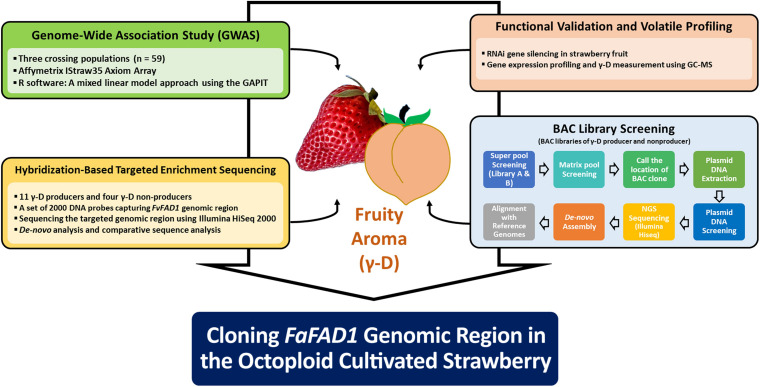
Schematic diagram for the genomic characterization of fruity aroma gene *FaFAD1* in the octoploid cultivated strawberry.

**FIGURE 2:**
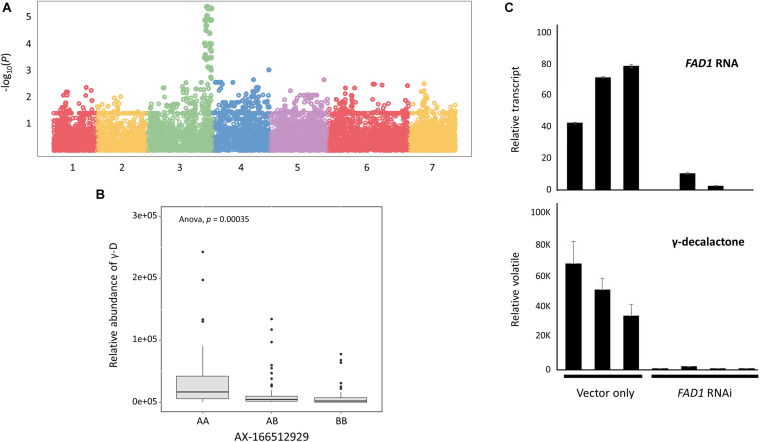
*FaFAD1* is a major locus that regulates for the production of γ-decalactone in the octoploid strawberry. **(A)** Manhattan plot of genome-wide association study for γ-decalactone. **(B)** Comparison of γ-decalactone content between different genotypes of a single marker (AX-166512929). **(C)** RNAi transient assay for *FaFAD1* in strawberry fruit. Bars represent mean, and error bars represent standard deviation for the three biological replicates.

To characterize the genomic region of *FaFAD1* in the octoploid cultivated strawberry, we conducted multiscale genomic approaches as shown in Figure 1. The locus controlling γ-D was previously identified on linkage group LGIII-2 (Sánchez-Sevilla et al., 2014). RNA-sequencing analysis identified a candidate gene, *FATTY ACID DESATURASE GENE 1* (*FaFAD1*), responsible for the presence/absence of γ-D in the octoploid strawberries (Chambers et al., 2014). To determine the physical location of the *FaFAD1* locus in the octoploid strawberry genome, IStraw35 whole genome SNP genotyping was conducted, with a genome-wide association study (GWAS) identifying a single peak for γ-D biosynthesis (*h*^2^ = 0.536) on chromosome group 3 (Figure 2A). To identify the subgenome harboring *FaFAD1*, 11 significant SNP probes were aligned to the four subgenomes of chromosome group 3 of the ‘Camarosa’ reference genome (Table 1). All of the 11 probes were located from 25.2 to 31.3 M on subgenome 3-2. Single-marker analysis with AX-166512929 that is closely associated with the *FaFAD1* locus suggests that this QTL is responsible for the natural variations of γ-D in the octoploid strawberry accessions (Figure 2B). There was also significant variation in γ-D content between homozygous (AA) and heterozygous (AB) accessions (Figure 2B).

**TABLE 1:** Physical location of the *FaFAD1* locus in diploid (*F. vesca*) and octoploid (*F.* × *ananassa*) strawberry.

SNP ID^*a*^	Chr. location (*F. vesca*)	Physical location (*F. vesca*)^*b*^	SNP marker location in *F.* × *ananassa* cv. Camarosa			
			
			Chr. 3-1	Chr. 3-2	Chr. 3-3	Chr. 3-4
AX-166512929	Chr. 3	30,211,312	−	29,761,869	−	2,002,493
AX-166504731	Chr. 3	32,524,288	−	30,719,612	−	893,136
AX-166510266	Chr. 3	29,399,062	−	29,191,207	−	3,572,806
AX-166517843	Chr. 3	29,567,447	2,270,475	29,384,352	−	−
AX-166504721	Chr. 3	32,313,444	807,512	30,650,836	−	965,986
AX-166522139	Chr. 3	32,370,296	−	30,668,160	−	−
AX-166512832	Chr. 3	32,424,673	−	30,684,817	−	987,060
AX-89912298	Chr. 3	29,593,040	2,262,160	29,406,849	27,457,717	2,555,020
AX-166519513	Chr. 3	32,812,041	96,868	31,307,920	29,608,552	155,496
AX-89912372	Chr. 3	31,060,850	1,221,378	30,397,639	−	1,385,503
AX-123524646	Chr. 3	24,023,662	6,600,875	25,184,632	23,414,517	6,539,416

*^a^Axiom 35K SNP marker.*

*^b^physical location shown in base pair (bp).*

Agroinfiltration experiments were used to test the requirement of *FAD1* for γ-D production. Developing fruits of a γ-D-producing genotype were treated with *Agrobacterium* cultures containing an RNAi construct targeting *FaFAD1*. Decreased accumulation of *FaFAD1* transcripts was related to a significant reduction in γ-D (Figure 2C). These results substantially provide evidence that the *FaFAD1* gene is a key regulator for the production of γ-D in the octoploid strawberry.

### A Large Deletion Was the Only Genomic Variation Linked to γ-D Production

The published allele for *FaFAD1* (Sánchez-Sevilla et al., 2014) was not detected in the ‘Camarosa’ reference genome, which is consistent with this cultivar not producing γ-D. Thus, it is not possible to examine the genomic structure of *FaFAD1* using the current reference genome for the octoploid strawberry. Using hybridization capture-based target enrichment sequencing, we investigated the 100 kb region of *FaFAD1* to examine for the potential genomic variants that could be linked to γ-D content. Hybridization probes for the genomic region of *FaFAD1* were designed using the *F. vesca* genome sequence. A total of 57,308,440 paired-end reads were generated for the target region of 100 kb, and *de novo* assembly was performed on 15 individuals (11 γ-D producers and four γ-D non-producers). A number of SNPs were found in the 100 kb region containing *FaFAD1*, but none correlated with the production of γ-D.

**FIGURE 3:**
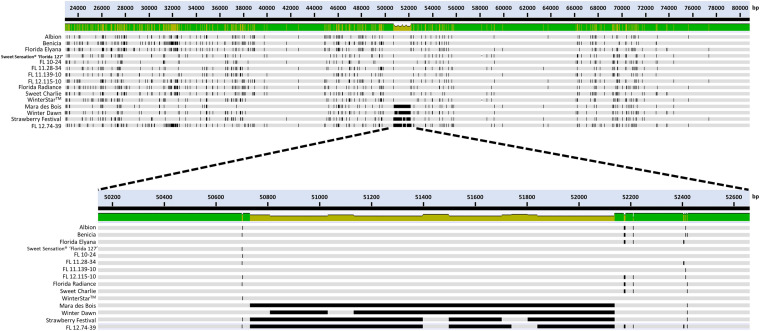
Hybrid-probe captured sequencing for the 100-kb flanking region of *FaFAD1* and comparative analysis among γ-D producers and γ-D non-producers. Illumina short reads (150 bp PE) were assembled to the *FvFAD1* region from the *F. vesca* reference genome (Edger et al., 2018). Each line represents a 100-kb flanking region of *FaFAD1* for 15 genotypes. The dark black lines represent deleted genomic sequences in the *FaFAD1* region.

The large deletion of *FaFAD1* occurred only in γ-D non-producers, including the genotypes ‘Winter Dawn’, ‘Mara des Bois’, ‘Strawberry Festival’, and FL 12.74-39 (Figure 3). In the *FaFAD1* genomic region, any CNVs associated with the *FaFAD1* gene did not present in all 11 γ-D producers. Because Illumina short reads (150 bp pair end) were used in this analysis, it was not possible to differentiate subgenome-specific sequence variations or determine the exact size of the insertion/deletion.

### The Deletion of *FaFAD1* Dictates γ-D Variation in the Octoploid Cultivated Strawberry

In order to exploit the genomic region of *FaFAD1* in γ-D-producing accessions, three BAC libraries developed from the University of Florida strawberry breeding accessions (FL 11.77-96 and ‘Florida Brilliance’: *FaFAD1*, and FL 14.101-225: *fafad1*) were screened with two gene-based markers, UFGDHRM5 (Noh et al., 2017) and qFaFAD1 (Sánchez-Sevilla et al., 2014) (Supplementary Tables 1, 3). Because both ‘Camarosa’ and ‘Reikou’ do not contain *FaFAD1*, the genomic structure could not be characterized from the reference sequence.

**FIGURE 4:**
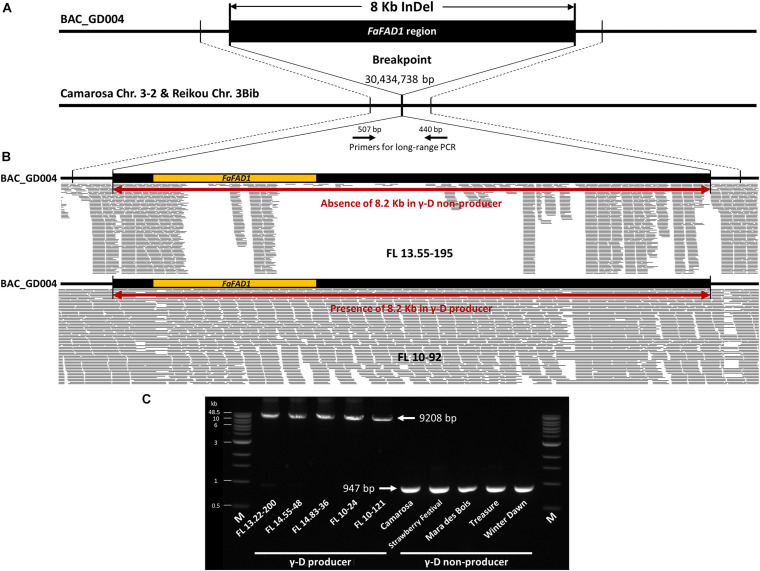
Detecting a deletion of *FaFAD1*. **(A)** Sequence alignment of a BAC clone, BAC_GD004, from library BB1 (‘Florida Brilliance’: γ-D producer) and corresponding regions of ‘Camarosa’ chromosome 3-2 and ‘Reikou’ haplotype 3Bib. An 8,262 bp deletion was detected in ‘Camarosa’ and ‘Reikou’, both γ-D non-producers. **(B)** Mapping Illumina sequence reads of γ-D producer (FL 10-92) and γ-D non-producer (FL 13.55-195) to BAC_GD004. **(C)** Long-range PCR results detect an 8,262 bp deletion between five γ-D producers and five γ-D non-producers. All PCR amplicons were similar to the expected size of 9,208 bp for γ-D producers and 947 bp for γ-D non-producers.

In the super pool (SP) screening using agarose gel electrophoresis with UFGDHRM5 and qFaFAD1 markers, 2–4 positive clones were identified from each of the BAC libraries, and further initiated to matrix pool (MP) screening for all libraries (Supplementary Figures 1, 2). In the result of MP screening, a total of six positive clones from two BAC libraries of γ-D producers, FL 11.77-96 and ‘Florida Brilliance’, were identified and further processed for sequencing. Illumina short reads from six BAC clones yielded approximately 7.7 million reads (average 149 bp length) and *de novo* assembly was performed (Supplementary Figure 3 and Supplementary Table 2). All of the six BAC clones generated 3–9 contigs with an average length of 135 kb. To determine sequence variations in the genomic region of *FaFAD1* among γ-D producers and γ-D non-producers, the contig sequences from all six BAC clones were aligned to the two octoploid reference sequences (cv. Camarosa and Reikou). A deletion of 8,262 bp, including 2,323 bp for *FaFAD1*, was consistently found in γ-D non-producers ‘Camarosa’ and ‘Reikou’ (Figure 4A and Supplementary Figure 4).

To confirm the difference in the genomic structure of the 8,262 bp indel region between γ-D producers and non-producers, whole genome sequencing reads of two breeding accessions (FL 10-92: γ-D producer, FL 13.55-195: γ-D non-producer) were mapped to the clone, BAC_GD004, from library BB1 (‘Florida Brilliance’) (Figure 4B). Illumina sequence reads were evenly mapped to the 8,262 bp insertion region in the γ-D producer, FL 10-92, while reads associated with *FaFAD1* were missing in the γ-D non-producer, FL 13.55-195. Some short reads were mapped to the flanking region of *FaFAD1* in the non-producer, because these reads are from other homeologous subgenomes of chromosome 3. The reads mapped to the promoter and coding region of *FaFAD1* are from the homologous gene located in chromosome 6. This homologous gene is not functionally associated with the production of γ-D production. Furthermore, to examine if the 8,262 bp presence/absence polymorphism is consistently present in other cultivated strawberry accessions, the long-range PCR was conducted with each of five γ-D producers and γ-D non-producers. All five γ-D producers tested had a PCR product of the expected size (9,209 bp), while 947 bp was amplified in other γ-D non-producers due to the absence of 8,262 bp (Figure 4C). Taken altogether, our results strongly indicate that the presence/absence variation of *FaFAD1* region is directly related to the production of γ-D in the octoploid strawberry.

### Codominant Marker Reveals Gene Dosage Effects on γ-D Production

**FIGURE 5:**
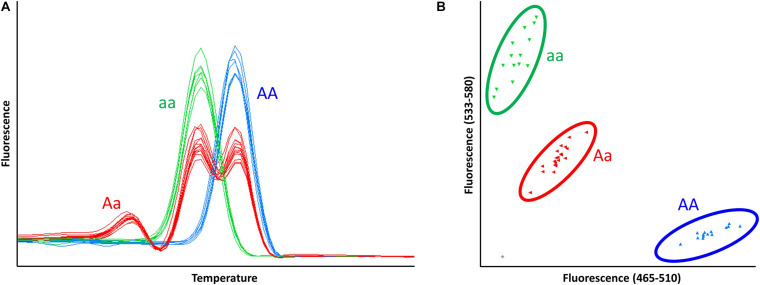
Differentiation of homo- and heterozygous γ-D producers using codominant HRM and KASP markers, NGD001 and UFGDKASP. **(A)** The pattern of HRM melting curve for HRM marker, NGD001, in 48 accessions. **(B)** Endpoint genotyping analysis of KASP marker, UFGDKASP, in 48 accessions. AA: homozygote γ-D producer, Aa: heterozygote γ-D producer, aa: homozygote γ-D non-producer.

It has been demonstrated that the content of γ-D varies among producers (Chambers et al., 2014). As shown in Table 2, the amount of γ-D in 48 accessions, 38 with *FaFAD1* and 10 accessions without *FaFAD1*, was measured using gas chromatography/mass spectroscopy (GC-MS) (Table 2). The content of γ-D is widely diverse among the 38 producers, while γ-D is not detectable in γ-D non-producers. To examine *FaFAD1* gene dosage effects on the variation in γ-D content, codominant high-resolution melting (HRM) and Kompetitive Allele Specific PCR (KASP) markers were developed from the flanking region of *FaFAD1* to amplify a common genomic sequence in both γ-D producers and γ-D non-producers. An 11-bp InDel located at 30,134,000 bp on chromosome 3-2 (‘Camarosa’) was targeted by HRM and KASP markers and tested on the 48 accessions (Figure 5, Supplementary Figure 4, and Supplementary Table 3). HRM and KASP markers developed from the flanking region of *FaFAD1* were used to amplify a common genomic sequence in both γ-D producers and non-producers to examine the *FaFAD1* gene dosage effects (Supplementary Figure 4).

**TABLE 2:** Markers genotype data and volatile content of γ-D in 48 accessions using HRM and KASP markers, and gas chromatography/mass spectrometry.

Accession	Female		Male	Source	NGD001	UFGDKASP	Area of Avg. γ-decalactone
FL 18.50-36	Florida Beauty	×	FL 15.89-25	University of Florida	AA	AA	1,906,279,564
FL 18.50-91	Florida beauty	×	FL 15.89-25	University of Florida	AA	AA	1,716,735,350
FL 18.50-96	Florida beauty	×	FL 15.89-25	University of Florida	AA	AA	1,450,960,595
FL 13.22-200	Sweet sensation^®^ “Florida 127”	×	FL 11.28-34	University of Florida	AA	AA	1,337,840,264
FL 18.50-52	Florida beauty	×	FL 15.89-25	University of Florida	AA	AA	1,333,444,656
FL 18.50-124	Florida beauty	×	FL 15.89-25	University of Florida	AA	AA	1,309,594,966
FL 14.55-48	FL 10-121	×	Sweet sensation^®^ “Florida 127”	University of Florida	AA	AA	1,167,611,378
FL 12.115-10	FL 10.133-98	×	FL 11.141-36	University of Florida	Aa	Aa	1,065,855,455
FL 10-24	FL 07-193	×	FL 07-168	University of Florida	AA	AA	1,015,450,185
FL 10-121	FL 07-68	×	FL 08-50	University of Florida	AA	AA	998,243,010
FL 11.31-54	WinterStar^TM^	×	FL 07-193	University of Florida	AA	AA	971,125,697
FL 14.83-36	FL 10-121	×	FL 07-193	University of Florida	AA	AA	951,911,108
Sweet Charlie	FL 80-456	×	Pajaro	University of Florida	Aa	Aa	752,825,948
FL 12.55-220	FL 10-47	×	Sweet sensation^®^ “Florida 127”	University of Florida	AA	AA	643,674,054
WinterStar^TM^	Florida Radiance	×	Earlibrite	University of Florida	Aa	Aa	629,417,671
FL 14.10-17	FL 11.77-96	×	FL 11.31-54	University of Florida	AA	AA	589,598,908
FL 15.56-134	FL 12.5-130	×	FL 10-121	University of Florida	Aa	Aa	588,413,724
FL 15.42-183	FL 10-46	×	FL 11.58-72	University of Florida	Aa	Aa	527,526,224
FL 12.75-77	FL 09-150	×	FL 09-100	University of Florida	Aa	Aa	508,672,061
Florida Brilliance	FL 11.31-14	×	FL 10-153	University of Florida	Aa	Aa	473,334,916
FL 13.27-142	FL 06-89	×	FL 10-47	University of Florida	Aa	Aa	464,913,451
FL 12.26-49	Sweet sensation^®^ “Florida 127”	×	FL 07-134	University of Florida	Aa	Aa	457,110,416
FL 11.71-9	FL 06-38	×	FL 07-193	University of Florida	AA	AA	438,927,503
Florida Beauty	AU 2010-119	×	Florida radiance	University of Florida	Aa	Aa	399,415,427
Sweet Sensation^®^ “Florida 127”	WinterStar^TM^	×	FL 02-58	University of Florida	Aa	Aa	389,982,544
FL 14.37-103	FL 10-24	×	FL 10-89	University of Florida	Aa	Aa	383,769,498
FL 12.93-4	FL 10-24	×	FL 11.107-5	University of Florida	Aa	Aa	364,489,979
FL 11.28-34	Florida radiance	×	FL 07-134	University of Florida	Aa	Aa	333,911,727
Florida Elyana	FL 96-114	×	FL 95-200	University of Florida	Aa	Aa	308,042,692
FL 15.76-45	FL 12.93-4	×	FL 12.55-220	University of Florida	Aa	Aa	305,626,231
FL 13.51-134	Sweet sensation^®^ “Florida 127”	×	FL 10-47	University of Florida	Aa	Aa	285,509,091
Florida Radiance	Winter dawn	×	FL 99-35	University of Florida	Aa	Aa	231,000,642
FL 14.55-203	FL 10-121	×	Sweet sensation^®^ “Florida 127”	University of Florida	Aa	Aa	180,351,763
FL 18.50-120	Florida Beauty	×	FL 15.89-25	University of Florida	Aa	Aa	84,966,825
FL 18.50-20	Florida beauty	×	FL 15.89-25	University of Florida	Aa	Aa	74,313,718
FL 18.50-49	Florida beauty	×	FL 15.89-25	University of Florida	Aa	Aa	71,291,037
FL 18.50-65	Florida beauty	×	FL 15.89-25	University of Florida	Aa	Aa	25,692,788
FL 18.50-42	Florida beauty	×	FL 15.89-25	University of Florida	Aa	Aa	23,038,727
FL 13.22-336	Sweet sensation^®^ “Florida 127”	×	FL 11.28-34	University of Florida	aa	aa	24,924,665
FL 12.74-39	FL 06-89	×	FL 11.63-41	University of Florida	aa	aa	24,864,174
FL 13.19-86	FL 06-134	×	FL 10-51	University of Florida	aa	aa	18,415,501
Fronteras	Unknown	×	Unknown	U. of California Davis	aa	aa	10,899,839
FL 13.55-195	FL 10-47	×	FL 10-153	University of Florida	aa	aa	7,760,551
Camarosa	Douglas	×	Cal. 85.218-605	U. of California Davis	aa	aa	−
Strawberry Festival	Rosa linda	×	OsoGrande	University of Florida	aa	aa	−
Treasure	A3	×	OsoGrande	Florida	aa	aa	−
Winter Dawn	FL 93-103	×	FL 95-316	University of Florida	aa	aa	−
Mara des Bois	Gento × Ostara	×	RedGaunlet × Korona	France	aa	aa	−

*AA, homozygote γ-D producer genotype; Aa, heterozygote γ-D producer genotype; aa, homozygote γ-D non-producer genotype;−, unknown.*

**FIGURE 6:**
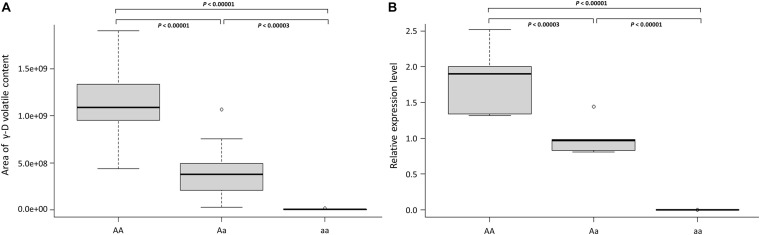
*FaFAD1* transcript dosage regulates γ-D content in the octoploid strawberry. **(A)** Distribution of γ-D volatile abundance per *FaFAD1* genotype. Boxplot showing the area of γ-D volatile content of each genotype for 48 accessions shown in Table 2. **(B)** Relative level of *FaFAD1* transcript by *FaFAD1* genotype. The boxplot represents gene expression distribution of each genotype, “AA” (FL 18.50-91, FL 18.50-52, FL 18.50-36, FL 18.50-124, and FL 18.50-96), “Aa” (FL 18.50-120, FL 18.50-49, FL 18.50-20, FL 18.50-42, and FL 18.50-65), and “aa” (‘Camarosa’, ‘Strawberry Festival’, ‘Mara des Bois’, ‘Treasure’, and ‘Winter Dawn’). AA: homozygote γ-D producer, Aa: heterozygote γ-D producer, aa: homozygote γ-D non-producer. The statistical significance of the differences was estimated using *t*-test. Boxplot: the black line in the middle of the boxes is the median, and the bottom and top of the boxes indicate the 25th and 75th percentiles. Whiskers (T-bars) are the minimum and maximum values. The empty dot is outlier. Both experiments **(A,B)** were done with three biological replicates and each replicate has at least three fruits.

All tested markers including a dominant marker (UFGDHRM5) detected the presence and absence of *FaFAD1* in the accessions (Figure 5 and Table 2). It was of interest to test if variation was associated with dosage imparted from the homo- or heterozygous state of the *FaFAD1* gene. The NGD001 and UFGDKASP markers distinguished between heterozygous (Aa) and homozygous (AA) genotypes among γ-D producers (Figure 5 and Table 2). The relative abundance of γ-D was significantly higher in homozygous than heterozygous producers (Figure 6A). Steady-state transcript levels for *FaFAD1* between the homozygous (AA) and heterozygous (Aa) γ-D producers were measured via qRT-PCR (Figure 6B). We found that transcript levels of the homozygous “AA” genotypes were significantly higher than the heterozygous “Aa” genotypes. The differences in transcript abundance match well with metabolite accumulation, indicating that the allele dosage is the cause for the variations of γ-D content in the octoploid cultivated strawberry.

## Discussion

Strawberry fruit has one of the most complex aromas and it has been reported that differences in aroma volatiles can improve strawberry fruit quality (Pelayo-Zaldivar et al., 2005; Dong et al., 2013). Several studies described the importance of key compounds of aroma production and the genetic loci or genes that control fruity aroma in peach (Eduardo et al., 2013; Pirona et al., 2013), apple (Dunemann et al., 2009; Costa et al., 2013), and octoploid strawberry (Aharoni et al., 2004; Zorrilla-Fontanesi et al., 2012). One of the key compounds, γ-decalactone, has a distinctly peach aroma, which increases the perception of sweetness in both peach (Zhang et al., 2010; Zhu and Xiao, 2019) and strawberry fruits (Schwieterman et al., 2014; Ulrich and Olbricht, 2016). While strawberries have a unique combination of sugars, acids, and volatile organic compounds (VOCs), some favorable flavors have been neglected due to the limited resources of strawberry breeding programs (Folta and Klee, 2016; Ulrich and Olbricht, 2016). In recent years, sensory qualities have become popular targets for genetic improvement, including qualities such as sweetness and unique flavors (Latrasse, 1991; Cruz-Rus et al., 2017). *FaFAD1* is a priority for these reasons, as it enhances the fruity flavor and sweetness of strawberries. This report and others have shown that the presence/absence and nature of the causal biosynthetic gene make γ-D an attractive target for marker-assisted breeding for enhanced flavor and aroma.

With the analysis of GWAS, a major locus, *FaFAD1*, controlling the production of γ-D volatile was identified at octoploid chromosome 3-2 (Table 1). Although the content of γ-D is controlled by one major locus (a candidate gene *FaFAD1*), the accumulation of γ-D varies widely (Larsen et al., 1992; Jetti et al., 2007; Zorrilla-Fontanesi et al., 2012; Chambers et al., 2014; Sánchez-Sevilla et al., 2014). These reports indicate that the physical presence/absence of the *FaFAD1* gene correlates with γ-D accumulation. In this report, the *FaFAD1* transcript was silenced using *Agroinfiltration* and RNAi in a γ-D-producing cultivar, demonstrating functionally that the gene is both necessary and sufficient for γ-D accumulation and that the volatile is not controlled by another element inside the deleted region.

To sequence the genomic region containing the functional *FaFAD1* allele, probe capture sequencing of the *FaFAD1* flanking region (100 kb) was performed on 11 γ-D producers and four γ-D non-producers. The multiple sequence alignment between the 15 genotypes evidently showed the absence of *FaFAD1* in γ-D non-producers (Figure 3). Our findings indicate that a single copy of *FaFAD1* located in chromosome 3 is responsible for production of γ-D.

Among polyploid genomes in general, high sequence similarity between homoeologous chromosomes is often a challenge in differentiating subgenomes (Mandáková et al., 2014). The subgenomes of polyploid plants are generally large and contain extensive repeats, which can greatly impede genome assembly resulting in non-contiguous and incorrect assemblies (Saski et al., 2017). Therefore, it remains challenging to assemble short-read sequences to specific subgenomes (Birchler and Veitia, 2012; Madlung and Wendel, 2013; Mandáková et al., 2014).

To overcome these difficulties, BAC-based physical maps combined with high-density genetic maps were used to improve the accuracy of whole-genome sequence assemblies (Luo et al., 2010; Ariyadasa and Stein, 2012; Sierro et al., 2013). Whole-genome sequencing and BAC libraries are physical and lasting genomic resources that have critical value as tools for positional cloning of genes and associated regulatory sequences (Saski et al., 2017). Here, the causal *FaFAD1* deletion region in the commercial octoploid strawberry was successfully characterized by using the BAC library sequencing approach (Figure 4).

In this study, approximately 100-kb *FaFAD1* flanking region of alleles was obtained through the six BAC clones. When comparing these BAC clones to the two reference genomes cv. Camarosa and Japanese cv. Reikou, which are γ-D non-producers, it was confirmed that there is an 8,262 bp deletion in subgenome 3-2 of ‘Camarosa’ and Ch3Bib of ‘Reikou’ (Figure 4A). Moreover, it was confirmed that γ-D non-producers have the same deletion size of 8,262 bp through long-range PCR using γ-D producers and γ-D non-producers (Figure 4C). While these results are confined to University of Florida cultivars, it is likely that the same deletion will be a useful diagnostic tool across strawberry varieties. The two newly developed codominant markers, NGD001 and UFGDKASP, are much improved from the previous dominant marker (Noh et al., 2017) in terms of accuracy and throughput and permit improved parental selection and rapid screening of progeny for homozygous plants likely to be higher γ-D producers. It would be more valuable to further examine the deletion in diverse strawberry accessions from different breeding programs.

Osborn et al. (2003) reported that the allele dosage is expected to relate to different levels of gene expression that affect target traits. Thus, it is highly possible that polyploids could increase the potential variation in their gene expression, which reflects in phenotypic variation. The inclusion of increased variation in dosage-regulated gene expression has become important for genetic studies in polyploid species (Garcia et al., 2013; Hackett et al., 2013; Endelman et al., 2018). To determine allele dosage effects in γ-D producer accessions, the content of γ-D volatile was measured by GC-MS in 48 advanced selections. The genotype results from the two codominant markers were strongly related to γ-D abundance among the 38 γ-D producers (Table 2). In addition, the expression level of *FaFAD1* was related to the zygosity of the gene (Figure 6). This result indicates that there is a dosage effect on γ-D production.

In summary, the precise location of *FaFAD1* was confirmed in chromosome 3-2 of the octoploid strawberry genome. By utilizing hybridization-based targeted enrichment sequencing and the BAC libraries, a major presence and absence polymorphism (8,262 bp) was substantially found within the *FaFAD1* locus, which is associated with γ-D production in the octoploid strawberry. This *FaFAD1*-containing deletion was present only in γ-D non-producers and directly responsible for lack of fruity aroma flavor. The newly developed codominant markers for *FaFAD1* from this study revealed unique information for the allele dosage effect on the content of γ-D. It provides the new evidence of allele dosage effect on volatile synthesis, suggesting that altering allele number can be a potential tactic for genetic improvement of fruit flavor. Our results provide a directly translatable resource for strawberry breeders and research communities, which will further facilitate the development of new strawberry cultivars with improved flavor.

## Materials and Methods

### Genome-Wide Association Study

For the GWAS of γ-decalactone, we used the three crossing populations (*n* = 59) that were derived from the crosses ‘Florida Elyana’ × ‘Mara de Bois’ (population 10.113), ‘Mara des Bois’ × ‘Florida Radiance’ (population 13.75), and ‘Strawberry Festival’ × ‘Winter Dawn’ (population 13.76) published in Barbey et al. (2020). GWAS for γ-decalactone was conducted via a mixed linear model approach using the GAPIT v2 package (Tang et al., 2016) in R software version 3.3.1. We consider GWAS associations significant at an alpha of 0.05 AFTER correction for FDR. SNP markers from the Affymetrix IStraw35 Axiom Array (Bassil et al., 2015; Verma et al., 2016) were mapped to the diploid *F. vesca* physical map, as available genetic maps in octoploid do not include a majority of the IStraw35 markers used in this study.

### Transient Assay

Transient expression in strawberry fruits by agroinfiltration was performed according to Hoffmann et al. (2006). *Agrobacterium tumefaciens* strain EH105 containing the RNAi vector was used to perform transient expression analysis and its effect on γ-D abundance in strawberry fruits. The culture was grown at 28°C overnight, and then the bacterial culture was resuspended in infection buffer (10 mM MgCl_2_, 100 μM acetosyringone, and 10 mM MES, pH 6.0) and shaken for 4 h at room temperature before infiltration of fruits. After the infection, the fruits were collected for gene expression and measurement of γ-D. For quantitative reverse transcription PCR (qRT-PCR), total RNA from the fruits was isolated as described by Pillet et al. (2017) and 1 μg of total RNA was used to synthesize the cDNA. For the qRT-PCR assay, a transcript corresponding to a conserved hypothetical protein FaCHP1 (Clancy et al., 2013) was used as a constitutive reference. The qRT PCR was run on an Applied Biosystems StepOnePlus Real-Time PCR System using StepOne Software (v2.0) (Applied Biosystems, Foster City, CA, United States). The qRT-PCR data were analyzed using the comparative CT method (ΔΔCT) following the manufacturer’s direction. The gene expression of *FaFAD1* was run with three technical replicates and repeated for at least three biological replicates.

### Hybridization-Based Targeted Enrichment Sequencing and Data Analysis

Targeted enrichment of *FaFAD1* genomic region was performed by hybrid capture-based next-generation sequencing (NGS) using a synthetic library consisting of a final set of 2,000 Axiom^®^ IStraw35 384HT array probes (Cronn et al., 2012; Verma et al., 2016). The capture oligonucleotides were 150 nt long and were designed to target 100 kb of *FvFAD1* (Fvb3: 31,039,796–31,149,795, *F*. *vesca* genome v2.0 a2). Probes were designed for covering the entire 100 kb genomic region of *FaFAD1* with 3 × coverage depth. The targeted capture sequencing libraries were constructed using Nextera Rapid Capture Custom Enrichment Kit. The captured sequence was quality controlled using Agilent 2100 Bioanalyzer. All sequencing was accomplished on the Illumina HiSeq 2000 using paired-end 100-bp reads following standard manufacturer protocols. Fifteen accessions were used for this experiment; 11 γ-D producers, ‘Albion’, ‘Benicia’, ‘Florida Elyana’, ‘Florida Radiance’, ‘Sweet Charlie’, Sweet Sensation^®^ ‘Florida 127’, ‘Winterstar’, (FL 10-24, FL 11.28-34, FL 11.139-10, and FL 12.115-10), and 4 γ-D non-producers, ‘Mara des Bois’, ‘Strawberry Festival’, ‘Winter Dawn’, and FL 12.74-39.

Raw FASTQ files were first checked using the FastQC tool^3^. Raw short reads from each capture library was mapped to a 100-kb region of *FvFAD1* downloaded from Genome Database for Rosaceae (GDR^4^) with CLC Genomics Workbench 11.0^5^, using the following parameters: match score 1, mismatch cost 2, cost of insertions and deletions = linear gap cost, insertion cost = 3, deletion cost = 3, length fraction = 0.8, and similarity fraction = 0.9. Consensus sequences were generated from all runs giving a total of 15 accession sequences, which were exported in fasta sequence. The consensus sequences were imported into Geneious 11.0.5^6^ and Multiple Sequence Alignment (MSA) was performed using the geneious alignment algorithm.

### BAC Library Construction and Screening

Three BAC libraries were prepared from the etiolated leaf tissues of three strawberry accessions, consisting of two γ-D producers (FL 11.77-96 and ‘Florida Brilliance’) and one γ-D non-producer (FL 14.101-225) (Supplementary Table 1). For the tissue etiolation, strawberry plants were covered with black plastic bags and kept in a greenhouse for 2 weeks. The etiolated young white leaf tissues were collected for the DNA extraction. The preparation of high-molecular-weight DNA and library construction was performed at Amplicon Express Inc. (Pullman, Washington). The *Bam*HI or *Hin*dIII digested genomic fragments were cloned into a BAC library vector. The recombinant vector was used to transform DH10B *Escherichia coli* competent cells (Invitrogen, CA, United States). The library was stored in 384-well plates filled with 50 μl freezing LB Lennox medium [36 mM K_2_HPO_4_, 1.7 mM sodium citrate, 0.4 mM MgSO_4_, 6.8 mM (NH_4_)_2_SO_4_, 4.4% glycerol, and 12.5 lg/ml chloramphenicol]. Plates were incubated for 18 h, replicated, and kept at −80°C for long-term storage. Two libraries *Hin*dIII and *Bam*HI for each accession comprise a total of 32,000 clones, which represent approximately 5 × octoploid strawberry genome coverage. Each library set comprised of 16 super pools (SPs) with individual SPs representing BAC DNA from 10,000 clones for each enzyme. Each SP was divided into 23 MPs each consisting of three plate pools (PPs), eight row pools (RPs), and 12 column pools (CPs). Each library contains approximately 30,000 clones with an average insert size of 150 kb. Three BAC libraries provide about 15 × genome coverage of the octoploid strawberry, respectively.

To identify BAC clones containing *FaFAD1*, two gene-specific markers, UFGDHRM5 (Noh et al., 2017) and qFaFAD1 (Sánchez-Sevilla et al., 2014), were tested for all three libraries (Supplementary Table 3). PCR reactions were performed with 16 SPs and 23 MPs with controls, following an online tool from Amplicon Express^7^. All PCR amplifications were performed in 5-μl reactions containing 2 × AccuStart^TM^ II PCR ToughMix^®^ (Quantabio, MA, United States), 1 × LC Green^®^ Plus melting dye (BioFire, UT, United States), 0.5 μM of each HRM primer set, and 0.5 μl of DNA. The reaction conditions were as follows: 95°C for 3 min, 45 cycles at 95°C for 10 sec, 45 cycles at 62°C for 10 sec, and 45 cycles at 72°C for 20 sec.

The plasmid DNA from the BAC clones was isolated using the QIAGEN plasmid midi kit (QIAGEN, Hilden, Germany). For the NGS, each DNA was sequenced by an Illumina high-throughput sequencer with 150-bp paired-end (PE) sequencing strategy. The obtained sequence reads from Illumina were assembled using the *de novo* assembly program from CLC Genomics Workbench, version 11.0^8^.

### Development and Validation of Functional Codominant Marker

The 48 accessions were used for marker development and validation. DNA extraction was performed using the simplified CTAB method with modifications (Noh et al., 2017). BAC-containing sequences from γ-D-producing accession (FL 11.77-96 and ‘Florida Brilliance’) were compared to the octoploid reference genome ‘Camarosa’, which does not produce γ-D. Primers were designed from the polymorphic sequences using IDT’s PrimerQuest Software (San Jose, CA, United States). PCR amplifications were performed in a 5-μl reaction containing 2 × AccuStart^TM^ II PCR ToughMix^®^ (Quantabio, MA, United States), 1 × LC Green^®^ Plus melting dye (BioFire, UT, United States), 0.5 μM of each HRM primer sets, and 1 μl of DNA. The PCR and HRM analysis were performed in a LightCycler^®^ 480 system II (Roche Life Science, Germany) using a program consisting of an initial denaturation at 95°C for 5 min; 45 cycles of denaturation at 95°C for 10 sec, annealing at 62°C for 10 sec, and extension at 72°C for 20 sec. After PCR amplification, the samples were heated to 95°C for 1 min and cooled to 40°C for 1 min. Melting curves were obtained by melting over the desired range (60–95°C) at a rate of 50 acquisitions per 1°C. Melting data were analyzed using the Melt Curve Genotyping and Gene Scanning Software (Roche Life Science, Germany). Analysis of HRM variants was based on differences in the shape of the melting curves and in *T*_*m*_ values.

KASP was designed using the 3CR Bioscience website^9^, and the genotyping assay was performed using a LightCycler^®^ 480 system II (Roche Life Science, Germany). KASP assays were conducted in 5 μl reactions with 50 ng of DNA template, 2 × PACE^TM^ Genotyping Master Mix (3CR Bioscience, United Kingdom), and PACE^TM^ Assay mix (UFGDKASP-allele-1-FAM, UFGDKASP-allele-2-HEX, and one reverse primer UFGDKASP-common) in a LightCycler^®^ 480 Multiwell 384-well plate. PCR optimization was carried out as follows: initial denaturation at 94°C for 15 min, a touch-down step followed by 10 cycles of 94°C for 20 sec, and 61°C decreasing 0.6°C per cycle to a final annealing/extension temperature of 55°C for 1 min, followed by 33 cycles of 94°C for 20 sec, annealing/extension temperature of 55°C for 1 min, with a final genotyping stage of 37°C for 1 min, a suitable temperature for KASP detection, and finally a plate reading at 37°C for 1 sec. KASP genotyping data were analyzed using Endpoint Genotyping Software (Roche Life Science, Germany).

### Long-Range PCR for the *FaFAD1* Region

Tissue samples were collected from five homozygous genotype “AA” accessions (FL 13.22-200, FL 14.55-48, FL 14.83-36, FL 10-24, and FL 10-121) and five “aa” accessions (‘Camarosa’, ‘Florida Festival’, ‘Mara des Bois’, ‘Treasure’, and ‘Winter Dawn’). The DNA was extracted following the simplified CTAB method with modifications (Noh et al., 2017). The primer was designed to have a PCR amplicon size of 9,208 bp in γ-D producers and 947 bp in γ-D non-producers. PCR amplifications were performed in a 10-μl reaction containing Accustart Long Range SuperMix (Quantabio, MA, United States), 0.5 μM of each HRM primer sets, and 2 μl of DNA. PCR was performed in a ProFlex PCR system (Applied Biosystems, United States) using a program consisting of an initial denaturation at 95°C for 3 min followed by 35 cycles of denaturation at 92°C for 30 sec, annealing at 65°C for 10 min, and final extension at 72°C for 10 min. PCR products were separated by agarose gel electrophoresis (1.0%), stained with SYBR^®^ Safe DNA gel stain (Invitrogen, Perth, WA) in TAE-Buffer (TRIS-Acetate-EDTA-buffer, pH 8), and visualized with UVP GelStudio Plus touch imaging system (Analytik Jena, AG Germany).

### Volatiles Analysis

The fruit of 48 strawberry accessions was selected in the field of the Gulf Coast Research and Education Center (GCREC) in Wimauma, Florida, during the 2017/2018 season. Harvest dates were December 20, 2017, January 10, 2018, and January 29, 2018. The experiment was done with three biological replicates. Each replicate has at least 6–8 fruits for each genotype. All fruits of the 48 accessions were analyzed for the volatile γ-D using GC-MS in the 2018 season. Fruit processing for volatile analysis was conducted as follows: 4–6 fully ripe, clean, and normal-shaped berries were harvested from each genotype, the calyx from each berry was removed, and approximately 25 g of flesh was collected. Each sample was frozen in liquid nitrogen and stored at −80°C until GC-MS analysis. Each sample was blended with an equal weight of saturated 35% NaCl solution. The volatile 3-hexanone was added as an internal standard to a final concentration of 1 ppm prior to blending. A total of 5 ml aliquots was taken from each sample and dispensed into 20 ml glass vials and sealed with magnetic crimp caps (Gerstel, Baltimore, MD, United States). Samples were frozen at −20°C until analysis by GC-MS.

For GC-MS, a 2 cm tri-phase SPME fiber (50/30 μm DVB/Carboxen/PDMS, Supelco, Bellefonte, PA, United States) was used to collect and concentrate volatiles prior to running on an Agilent 6890 GC coupled with a 5973 N MS detector (Agilent Technologies, Palo Alto, CA, United States). Before analysis, samples were held at 4°C in a Peltier cooling tray attached to a MPS2 autosampler (Gerstel). An authentic γ-D standard (Sigma Aldrich, St. Louis, MO, United States) was run under the same chromatographic conditions as berry samples for verification of volatile identification. The area of each γ-D peak was normalized to the peak area of the internal standard, and normalized peak areas were compared between samples.

### Quantitative Real-Time PCR Analysis

For total RNA extraction, five homozygous “AA” genotypes (FL 18.50-91, FL 18.50-52, FL 18.50-36, FL 18.50-124, and FL 18.50-96) and five heterozygote “Aa” genotypes (FL 18.50-120, FL 18.50-49, FL 18-50-20, FL 18.50-42, and FL 18.50-65) were collected from a FL 18.50 advanced selection population. ‘Camarosa’, ‘Strawberry Festival’, ‘Mara des Bois’, ‘Treasure’, and ‘Winter Dawn’ with homozygote genotype “aa” were included as the reference genotype of γ-D non-producer. The experiment was performed with three biological replicates, and each replicate contains at least 5–8 fruits. Total RNA was extracted according to the protocol of the Spectrum^TM^ Plant Total RNA Kit (Sigma-Aldrich, St. Louis, MO, United States) and resuspended in a total volume of 50 μl of RNase-free water. cDNA synthesis was performed with the Luna Script RT Super Mix Kit (New England Biolabs. Ipswich, MA). Primer sequence of *FaFAD1*, GD_Rt1, was obtained from NCBI (GeneBank Accession: KF887973.1). Strawberry *GAPDH* gene (GeneBank Accession: AB363963.1), FaGAPDH2, was selected as an endogenous control (Supplementary Table 3). qRT-PCRs were carried out with a LightCycler^®^ 480 system II (Roche Life Science, Germany) using Forget-Me-Not^TM^ qPCR Master Mix (Biotium, Corporate Place Hayward, CA, United States). The PCR contents were carried out in a total volume of 5 μl, which contained 1 μl of template cDNA, 0.2 μl of each primer, 2.5 μl of EvaGreen qPCR Master Mix, and 1.1 μl of double-distilled H_2_O. The reaction conditions were 95°C for 5 min, followed by 40 cycles of 95°C for 20 sec, 60°C for 20 sec, and 72°C for 20 sec. This was followed by melting curve analysis, which was performed to confirm that each amplicon has a single product. The qRT-PCR experiment was repeated with at least three technical replications for each biological replicate. Relative fold difference was calculated by using the 2^−ΔΔCt^ method (Paolacci et al., 2009).

### Data Analysis and Experimental Design

For all comparisons of means among genotypes (AA, Aa, and aa), *t*-test was performed using R software version 3.3.1 (R Core Team, 2017). The procedures and strategies of this study for characterizing the *FaFAD1* region are summarized in Figure 1.

## Data Availability Statement

The datasets presented in this study can be found in online repositories. The names of the repository/repositories and accession number(s) can be found in the article/Supplementary Material. The sequence data of BAC_GD004 is available at this NCBI website (https://www.ncbi.nlm.nih.gov/, accession number MW584663).

## Author Contributions

YO performed the DNA extractions, bioinformatics study, HRM and KASP marker development, volatile analysis, and data analysis, and drafted the manuscript. CRB performed genome-wide association study, bioinformatics study, and data analysis. SC performed hybridization-based capture sequencing. ZF, JB, and AP participated in volatile analysis. KMF and JP conceived and performed in the transient assay. VMW participated in its design and coordination. SL conceived of the study, data analysis, and drafted the manuscript. All authors have read and approved the final manuscript.

## Conflict of Interest

The authors declare that the research was conducted in the absence of any commercial or financial relationships that could be construed as a potential conflict of interest. The reviewer IA declared a past co-authorship with several of the authors YO, VMW to the handling editor.

## Footnotes

1 https://www.rosaceae.org/

2 http://strawberry-garden.kazusa.or.jp/index.html

3 https://www.bioinformatics.babraham.ac.uk/projects/fastqc/

4 https://www.rosaceae.org/species/fragaria_vesca/genome_v4.0.a1

5 https://www.qiagenbioinformatics.com/

6 http://www.geneious.com/

7 http://puffer.ampliconexpress.com/

8 www.qiagenbioinformatics.com

9 https://3crbio.com/free-assay-design/

## Supplementary Material

Supplementary Figure 1 Super pool screening of BAC libraries AH3, BB1, and BH3 with two gene based markers, qFaFAD1 and UFGDHRM5. The numbers 1–16 indicate each superpool and “+,” “−” indicate positive and negative control. A yellow circle indicates a positive clone. AH3: BAC screening from AH3 SP1-16 using primers qFaFAD1 and UFGDHRM5.

Supplementary Figure 2 Matrix pool screening of positive clones from each super pools with two gene based markers, qFaFAD1 and UFGDHRM5, and location of positive clone. The number and position of BAC clone is determined by the combination of positive bands in each wells of plate, row, and column.

Supplementary Figure 3 DNA sequence alignment of six BAC clones. Green bars and blue box show the *FaFAD1* deletion region. Red bars show the *FaFAD1* primer sites of qFaFAD1 and UFGDHRM5.

Supplementary Figure 4 DNA sequence alignments of ‘Camarosa’, ‘Reikou’, and BAC_GD004. The positions of HRM and KASP markers are highlighted with a yellow box and green color, respectively. A blue box indicates the 8,262 bp deletion between γ-D producers and γ-D non-producers. SNPs and InDels were highlighted gray and red colors, respectively.

Supplementary Table 1 Bacterial Artificial Chromosome (BAC) libraries of three strawberry accessions.

Supplementary Table 2 *De novo* assembly results of six BAC clones.

Supplementary Table 3 Marker sequence information used in this study.
