# Supplementary material for: Genomic Characterization of the Fruity Aroma Gene, FaFAD1, Reveals a Gene Dosage Effect on γ-Decalactone Production in Strawberry (Fragaria × ananassa)
Source: Front Plant Sci. 2021 May 4;12:639345. doi: 10.3389/fpls.2021.639345 (PMC8129584; doi:10.3389/fpls.2021.639345)
Supplement: Supplementary Figure 1 — Super pool screening of BAC libraries AH3, BB1, and BH3 with two gene based markers, qFaFAD1 and UFGDHRM5. The numbers 1–16 indicate each superpool and “+,” “−” indicate positive and negative control. A yellow circle indicates a positive clone. AH3: BAC screening from AH3 SP1-16 using primers qFaFAD1 and UFGDHRM5. [file Presentation_1.PPTX]

## Slide 1
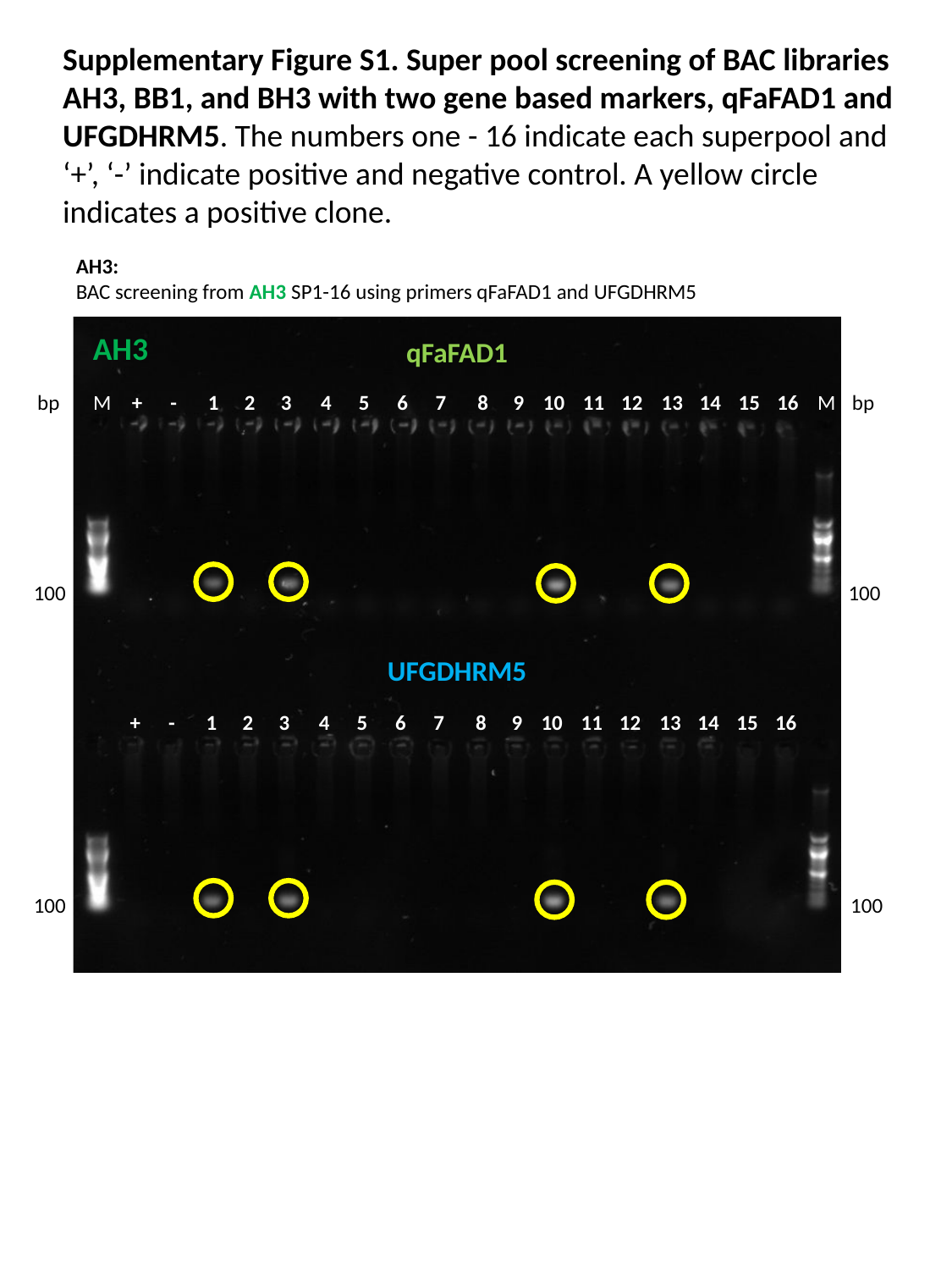

Supplementary Figure S1. Super pool screening of BAC libraries AH3, BB1, and BH3 with two gene based markers, qFaFAD1 and UFGDHRM5. The numbers one - 16 indicate each superpool and ‘+’, ‘-’ indicate positive and negative control. A yellow circle indicates a positive clone.
# AH3: BAC screening from AH3 SP1-16 using primers qFaFAD1 and UFGDHRM5
AH3
qFaFAD1
+
 -
1
2
3
4
5
6
7
8
9
10
11
12
13
14
15
16
bp
100
M
M
bp
100
UFGDHRM5
+
 -
1
2
3
4
5
6
7
8
9
10
11
12
13
14
15
16
100
100

## Slide 2
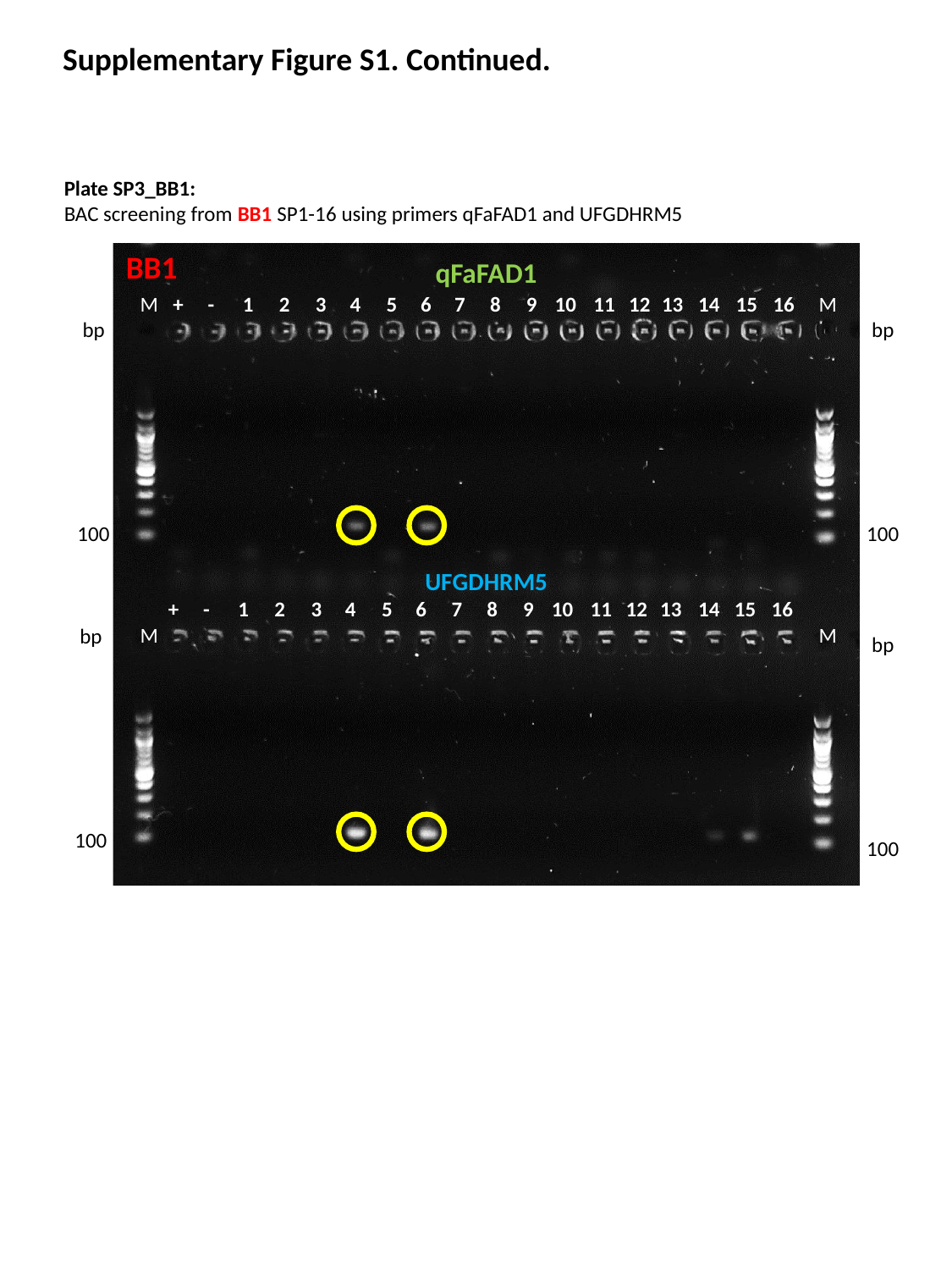

Supplementary Figure S1. Continued.
Plate SP3_BB1:
BAC screening from BB1 SP1-16 using primers qFaFAD1 and UFGDHRM5
BB1
qFaFAD1
+
 -
1
2
3
4
5
6
7
8
9
10
11
12
13
14
15
16
M
M
bp
bp
100
100
UFGDHRM5
+
 -
1
2
3
4
5
6
7
8
9
10
11
12
13
14
15
16
M
M
bp
bp
100
100
+
 -
2
3
4
5
6
7
8
9
10
11
12
13
14
15
16
1

## Slide 3
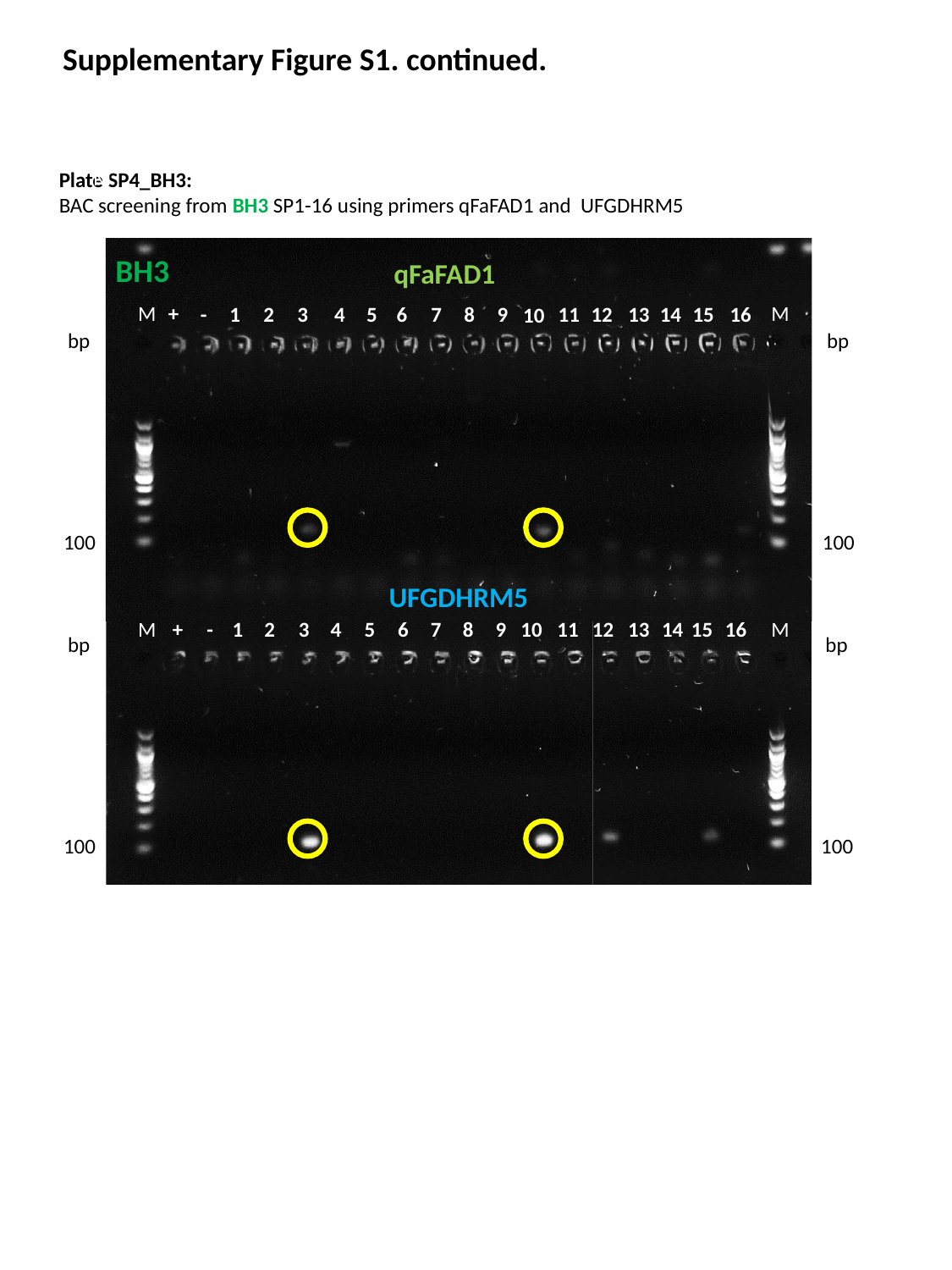

Supplementary Figure S1. continued.
M
Plate SP4_BH3:
BAC screening from BH3 SP1-16 using primers qFaFAD1 and UFGDHRM5
BH3
qFaFAD1
+
 -
1
2
3
4
5
6
7
8
9
11
12
13
14
15
16
10
M
M
bp
bp
100
100
UFGDHRM5
M
+
-
1
2
3
4
5
6
7
8
9
10
11
12
13
14
15
16
M
bp
bp
100
100

## Slide 4
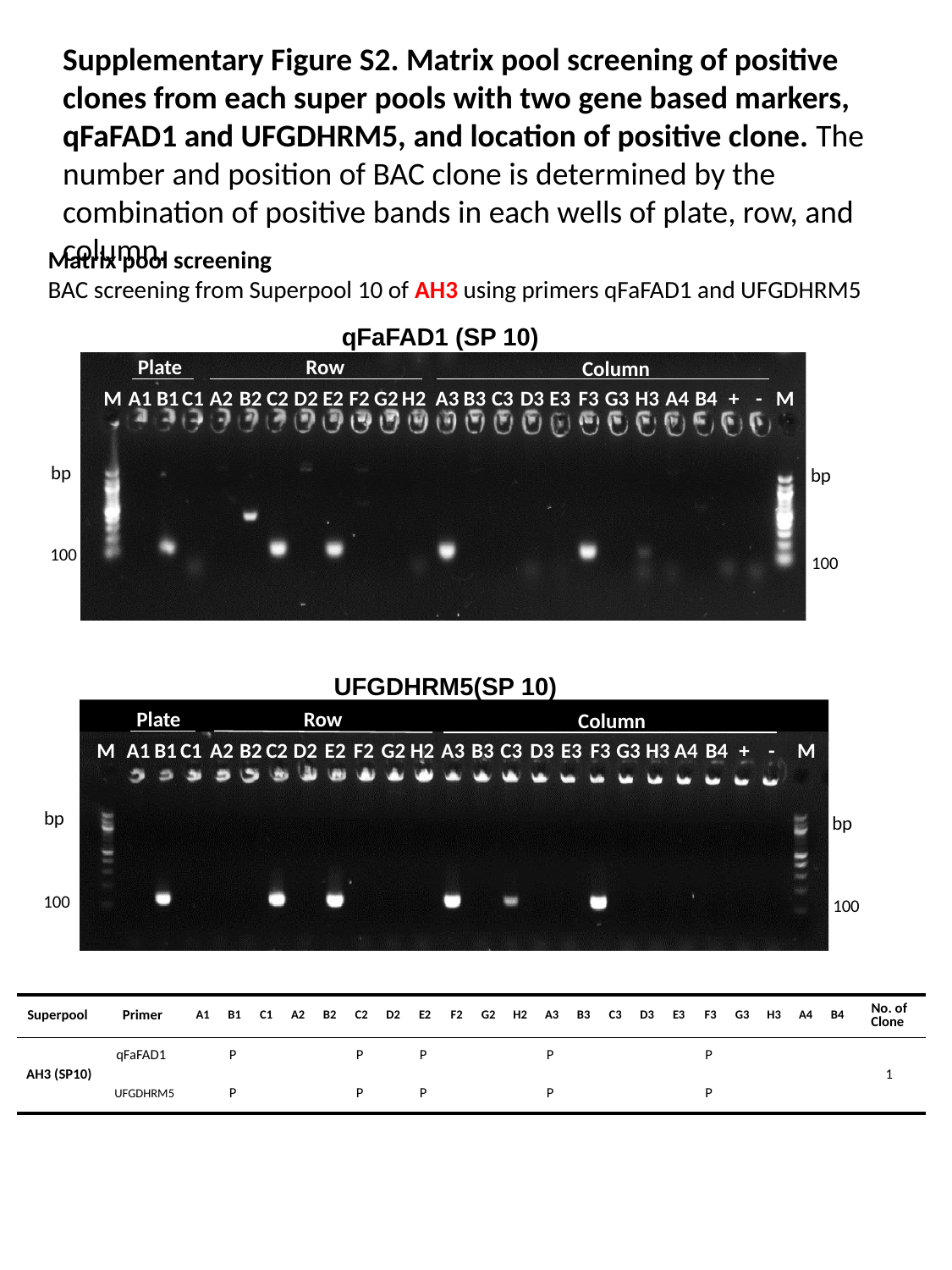

Supplementary Figure S2. Matrix pool screening of positive clones from each super pools with two gene based markers, qFaFAD1 and UFGDHRM5, and location of positive clone. The number and position of BAC clone is determined by the combination of positive bands in each wells of plate, row, and column.
Matrix pool screening
BAC screening from Superpool 10 of AH3 using primers qFaFAD1 and UFGDHRM5
qFaFAD1 (SP 10)
Plate
Row
Column
M
A1
B1
C1
A2
B2
C2
D2
E2
F2
G2
H2
A3
B3
C3
D3
E3
F3
G3
H3
A4
B4
+
-
M
bp
100
bp
100
UFGDHRM5(SP 10)
Plate
Row
Column
M
A1
B1
C1
A2
B2
C2
D2
E2
F2
G2
H2
A3
B3
C3
D3
E3
F3
G3
H3
A4
B4
+
-
M
bp
100
bp
100
| Superpool | Primer | A1 | B1 | C1 | A2 | B2 | C2 | D2 | E2 | F2 | G2 | H2 | A3 | B3 | C3 | D3 | E3 | F3 | G3 | H3 | A4 | B4 | No. of Clone |
| --- | --- | --- | --- | --- | --- | --- | --- | --- | --- | --- | --- | --- | --- | --- | --- | --- | --- | --- | --- | --- | --- | --- | --- |
| AH3 (SP10) | qFaFAD1 | | P | | | | P | | P | | | | P | | | | | P | | | | | 1 |
| | UFGDHRM5 | | P | | | | P | | P | | | | P | | | | | P | | | | | |

## Slide 5
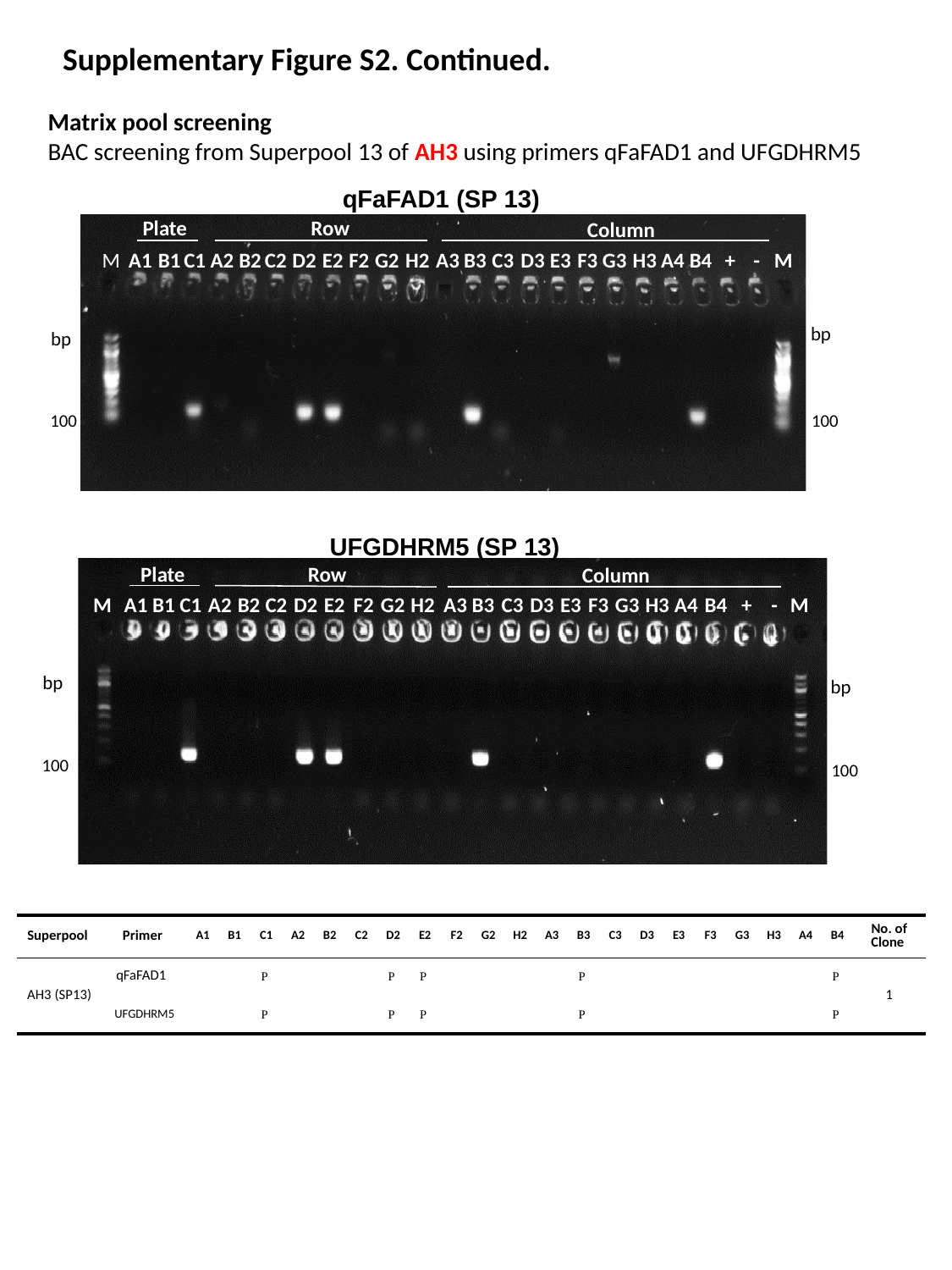

Supplementary Figure S2. Continued.
Matrix pool screening
BAC screening from Superpool 13 of AH3 using primers qFaFAD1 and UFGDHRM5
qFaFAD1 (SP 13)
Plate
Row
Column
M
A1
B1
C1
A2
B2
C2
D2
E2
F2
G2
H2
A3
B3
C3
D3
E3
F3
G3
H3
A4
B4
+
-
M
bp
100
bp
100
UFGDHRM5 (SP 13)
Plate
Row
Column
M
A1
B1
C1
A2
B2
C2
D2
E2
F2
G2
H2
A3
B3
C3
D3
E3
F3
G3
H3
A4
+
-
M
B4
bp
100
bp
100
| Superpool | Primer | A1 | B1 | C1 | A2 | B2 | C2 | D2 | E2 | F2 | G2 | H2 | A3 | B3 | C3 | D3 | E3 | F3 | G3 | H3 | A4 | B4 | No. of Clone |
| --- | --- | --- | --- | --- | --- | --- | --- | --- | --- | --- | --- | --- | --- | --- | --- | --- | --- | --- | --- | --- | --- | --- | --- |
| AH3 (SP13) | qFaFAD1 | | | P | | | | P | P | | | | | P | | | | | | | | P | 1 |
| | UFGDHRM5 | | | P | | | | P | P | | | | | P | | | | | | | | P | |

## Slide 6
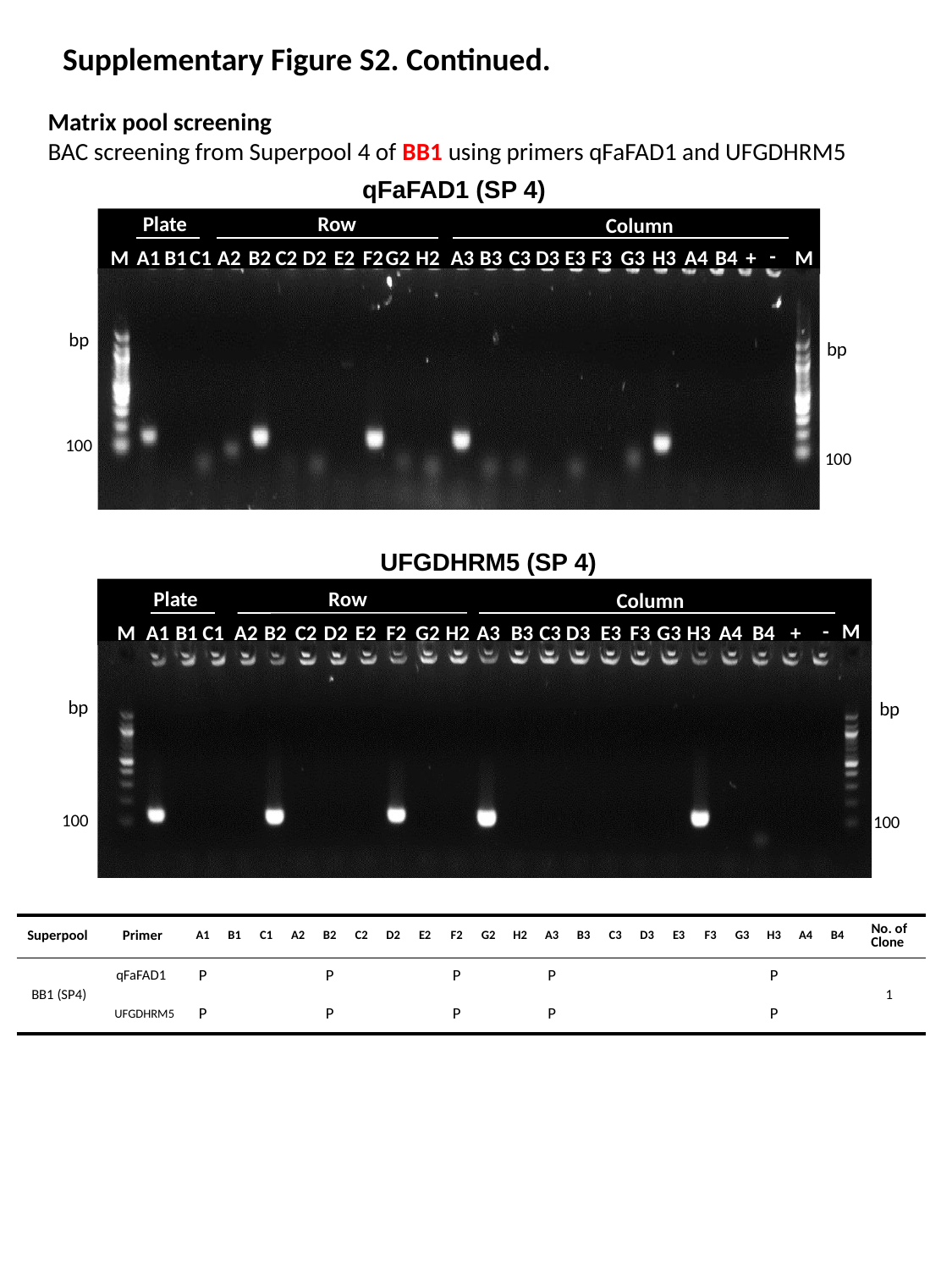

Supplementary Figure S2. Continued.
Matrix pool screening
BAC screening from Superpool 4 of BB1 using primers qFaFAD1 and UFGDHRM5
qFaFAD1 (SP 4)
Plate
Row
Column
-
M
A1
B1
C1
A2
B2
C2
D2
E2
F2
G2
H2
A3
B3
C3
D3
E3
F3
G3
H3
A4
B4
+
M
bp
100
bp
100
UFGDHRM5 (SP 4)
Plate
Row
Column
 -
M
M
A1
B1
C1
A2
B2
C2
D2
E2
F2
G2
H2
A3
B3
C3
D3
E3
F3
G3
H3
A4
B4
+
bp
100
bp
100
| Superpool | Primer | A1 | B1 | C1 | A2 | B2 | C2 | D2 | E2 | F2 | G2 | H2 | A3 | B3 | C3 | D3 | E3 | F3 | G3 | H3 | A4 | B4 | No. of Clone |
| --- | --- | --- | --- | --- | --- | --- | --- | --- | --- | --- | --- | --- | --- | --- | --- | --- | --- | --- | --- | --- | --- | --- | --- |
| BB1 (SP4) | qFaFAD1 | P | | | | P | | | | P | | | P | | | | | | | P | | | 1 |
| | UFGDHRM5 | P | | | | P | | | | P | | | P | | | | | | | P | | | |

## Slide 7
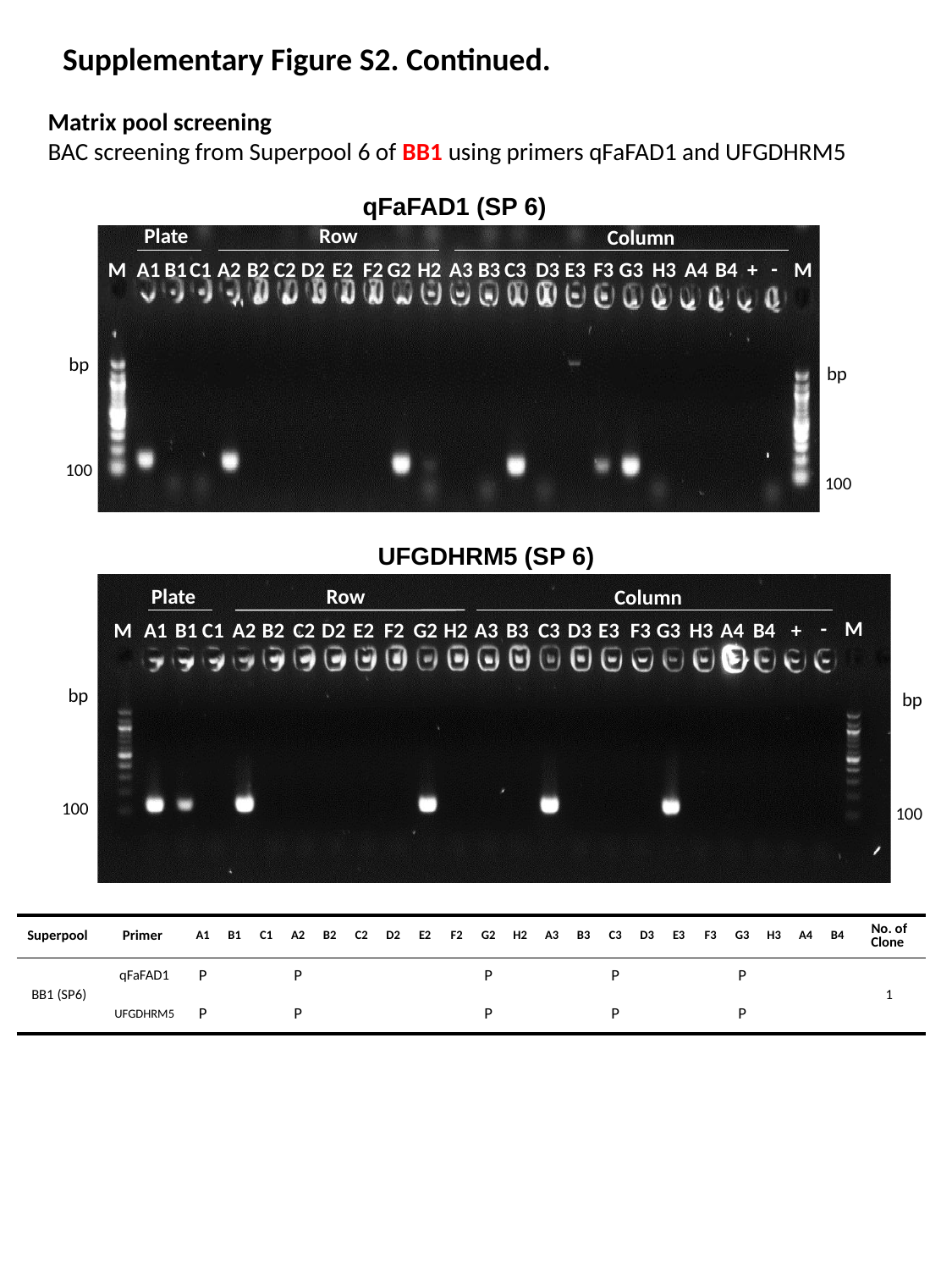

Supplementary Figure S2. Continued.
Matrix pool screening
BAC screening from Superpool 6 of BB1 using primers qFaFAD1 and UFGDHRM5
qFaFAD1 (SP 6)
Plate
Row
Column
-
M
A1
B1
C1
A2
B2
C2
D2
E2
F2
G2
H2
A3
B3
C3
D3
E3
F3
G3
H3
A4
B4
+
M
bp
100
bp
100
UFGDHRM5 (SP 6)
Plate
Row
Column
 -
M
M
A1
B1
C1
A2
B2
C2
D2
E2
F2
G2
H2
A3
B3
C3
D3
E3
F3
G3
H3
A4
B4
+
bp
100
bp
100
| Superpool | Primer | A1 | B1 | C1 | A2 | B2 | C2 | D2 | E2 | F2 | G2 | H2 | A3 | B3 | C3 | D3 | E3 | F3 | G3 | H3 | A4 | B4 | No. of Clone |
| --- | --- | --- | --- | --- | --- | --- | --- | --- | --- | --- | --- | --- | --- | --- | --- | --- | --- | --- | --- | --- | --- | --- | --- |
| BB1 (SP6) | qFaFAD1 | P | | | P | | | | | | P | | | | P | | | | P | | | | 1 |
| | UFGDHRM5 | P | | | P | | | | | | P | | | | P | | | | P | | | | |

## Slide 8
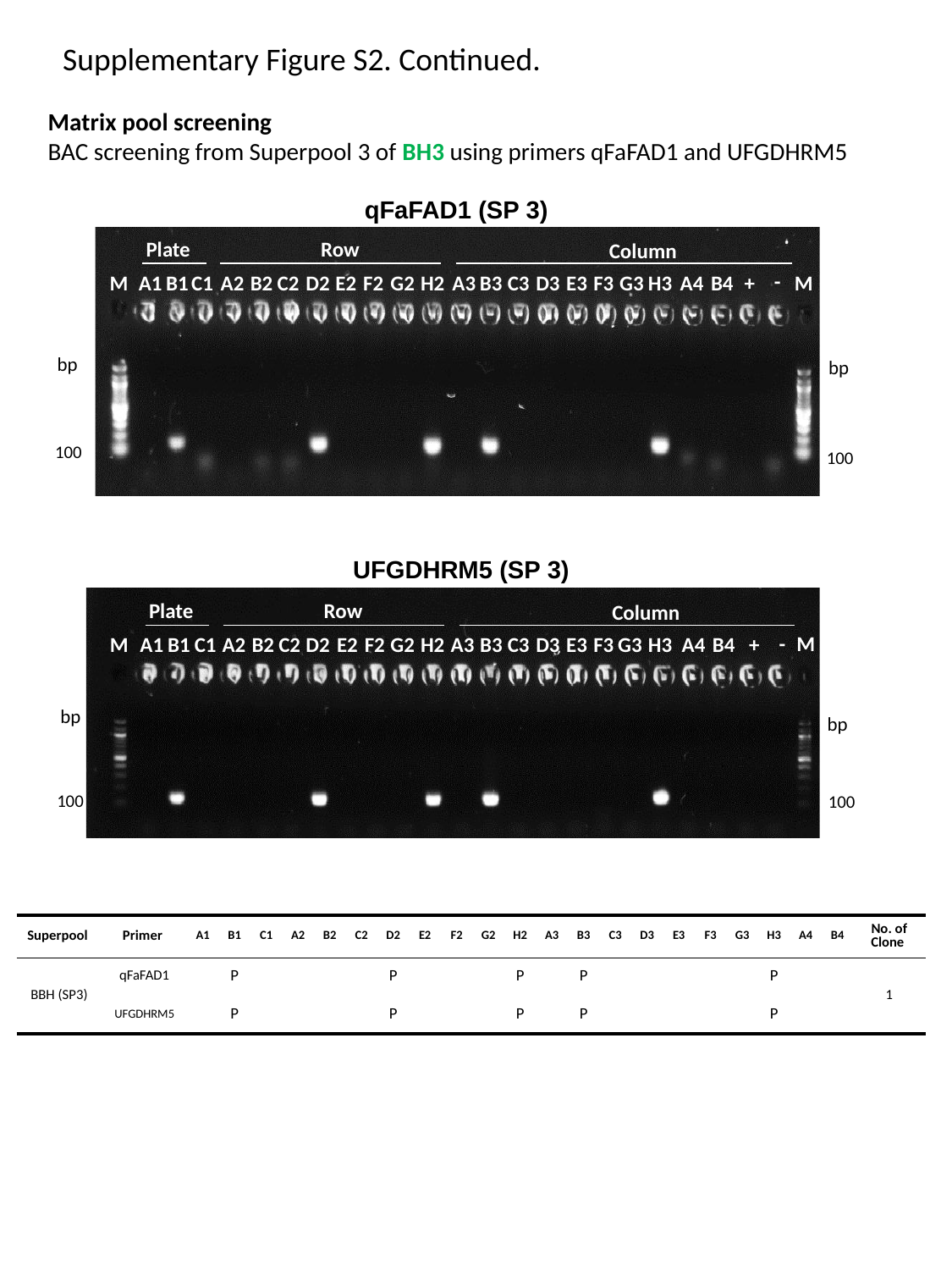

Supplementary Figure S2. Continued.
Matrix pool screening
BAC screening from Superpool 3 of BH3 using primers qFaFAD1 and UFGDHRM5
qFaFAD1 (SP 3)
Plate
Row
Column
-
M
A1
B1
C1
A2
B2
C2
D2
E2
F2
G2
H2
A3
B3
C3
D3
E3
F3
G3
H3
A4
B4
+
M
bp
100
bp
100
UFGDHRM5 (SP 3)
Plate
Row
Column
 -
M
M
A1
B1
C1
A2
B2
C2
D2
E2
F2
G2
H2
A3
B3
C3
D3
E3
F3
G3
H3
A4
B4
+
bp
100
bp
100
| Superpool | Primer | A1 | B1 | C1 | A2 | B2 | C2 | D2 | E2 | F2 | G2 | H2 | A3 | B3 | C3 | D3 | E3 | F3 | G3 | H3 | A4 | B4 | No. of Clone |
| --- | --- | --- | --- | --- | --- | --- | --- | --- | --- | --- | --- | --- | --- | --- | --- | --- | --- | --- | --- | --- | --- | --- | --- |
| BBH (SP3) | qFaFAD1 | | P | | | | | P | | | | P | | P | | | | | | P | | | 1 |
| | UFGDHRM5 | | P | | | | | P | | | | P | | P | | | | | | P | | | |

## Slide 9
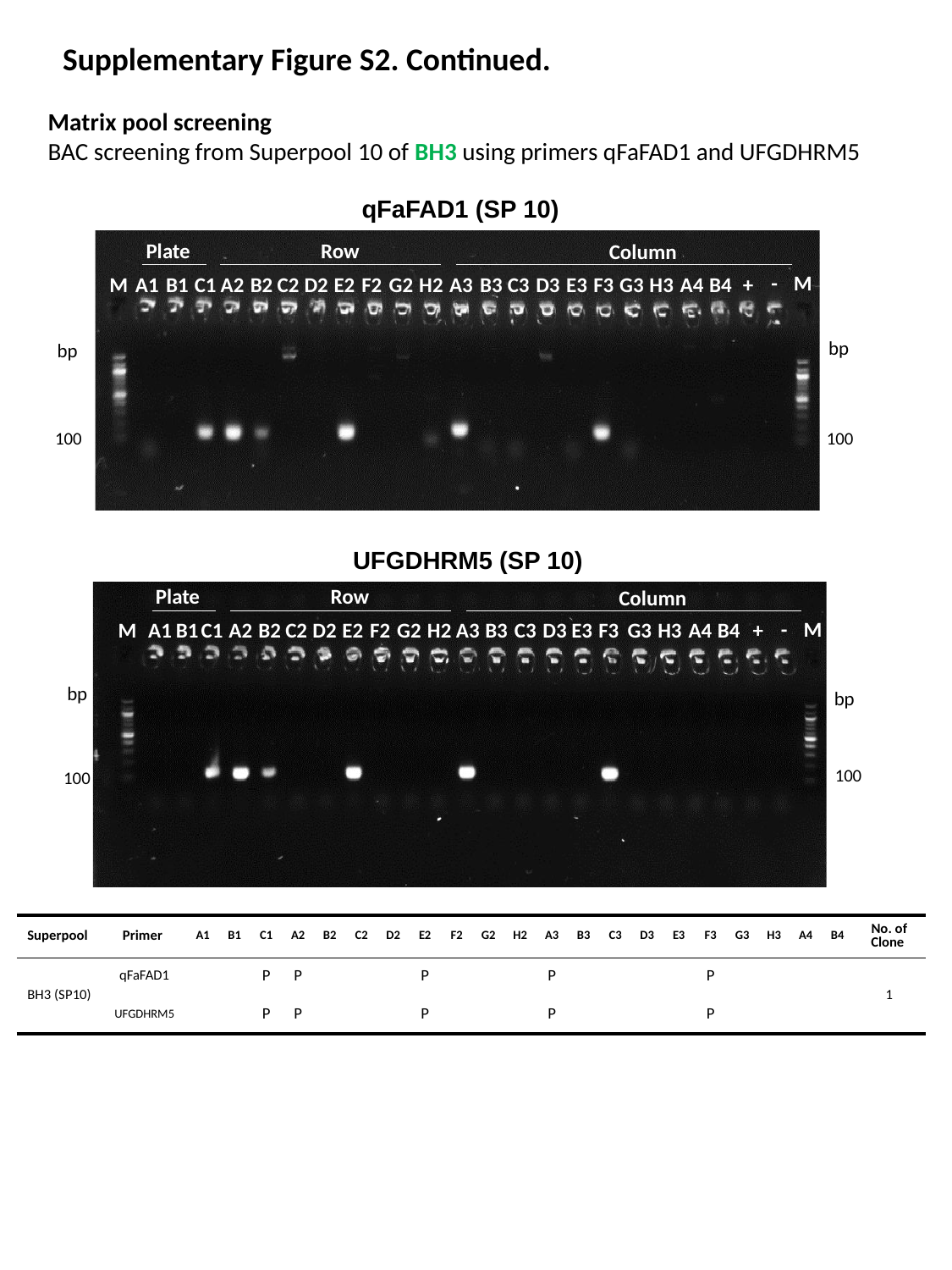

Supplementary Figure S2. Continued.
Matrix pool screening
BAC screening from Superpool 10 of BH3 using primers qFaFAD1 and UFGDHRM5
qFaFAD1 (SP 10)
Plate
Row
Column
-
M
M
A1
B1
C1
A2
B2
C2
D2
E2
F2
G2
H2
A3
B3
C3
D3
E3
F3
G3
H3
A4
B4
+
bp
100
bp
100
UFGDHRM5 (SP 10)
Plate
Row
Column
-
M
M
A1
B1
C1
A2
B2
C2
D2
E2
F2
G2
H2
A3
B3
C3
D3
E3
F3
G3
H3
A4
B4
+
bp
100
bp
100
| Superpool | Primer | A1 | B1 | C1 | A2 | B2 | C2 | D2 | E2 | F2 | G2 | H2 | A3 | B3 | C3 | D3 | E3 | F3 | G3 | H3 | A4 | B4 | No. of Clone |
| --- | --- | --- | --- | --- | --- | --- | --- | --- | --- | --- | --- | --- | --- | --- | --- | --- | --- | --- | --- | --- | --- | --- | --- |
| BH3 (SP10) | qFaFAD1 | | | P | P | | | | P | | | | P | | | | | P | | | | | 1 |
| | UFGDHRM5 | | | P | P | | | | P | | | | P | | | | | P | | | | | |

## Slide 10
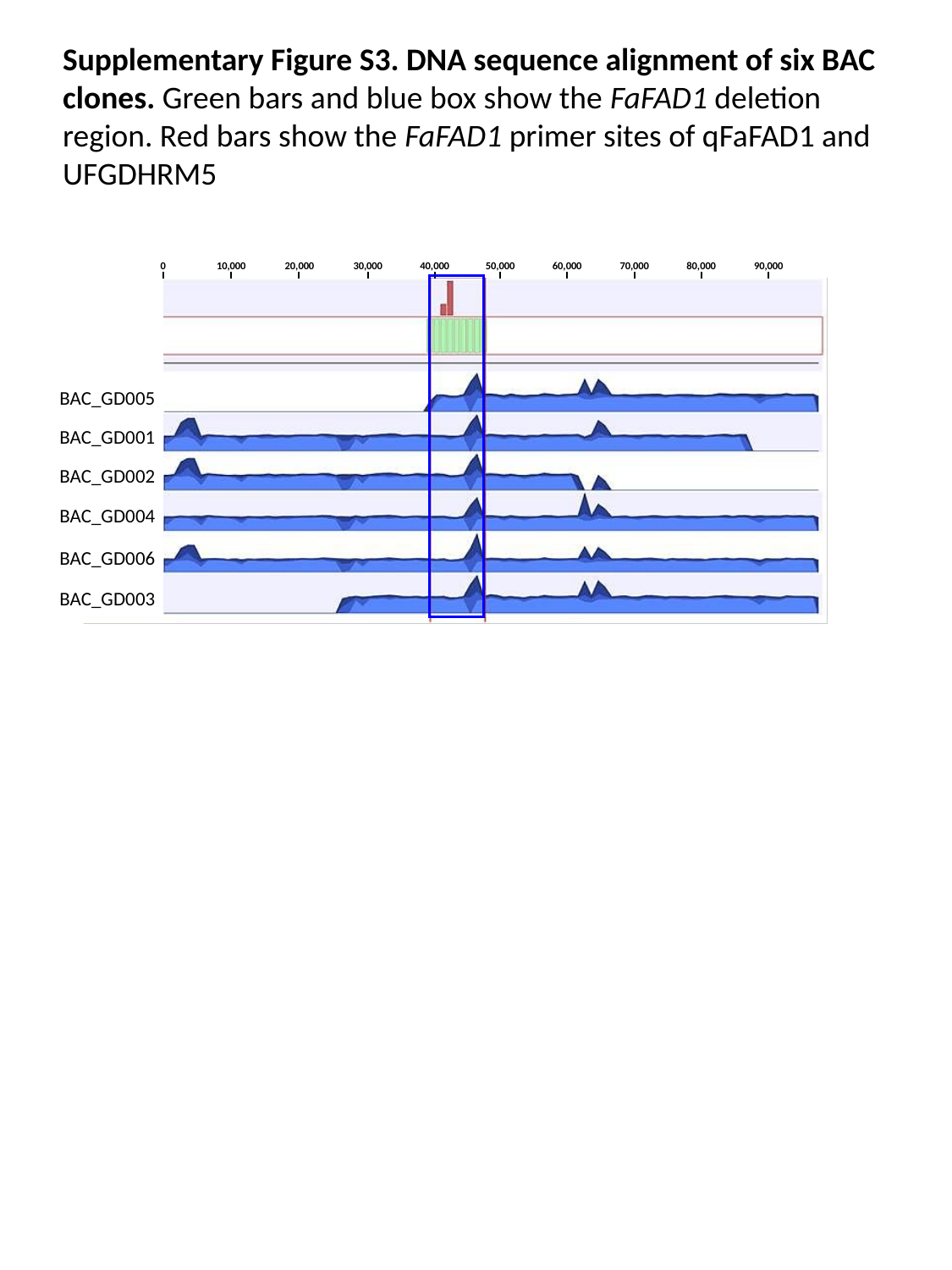

Supplementary Figure S3. DNA sequence alignment of six BAC clones. Green bars and blue box show the FaFAD1 deletion region. Red bars show the FaFAD1 primer sites of qFaFAD1 and UFGDHRM5
0
10,000
20,000
30,000
40,000
50,000
60,000
70,000
80,000
90,000
BAC_GD005
BAC_GD001
BAC_GD002
BAC_GD004
BAC_GD006
BAC_GD003

## Slide 11
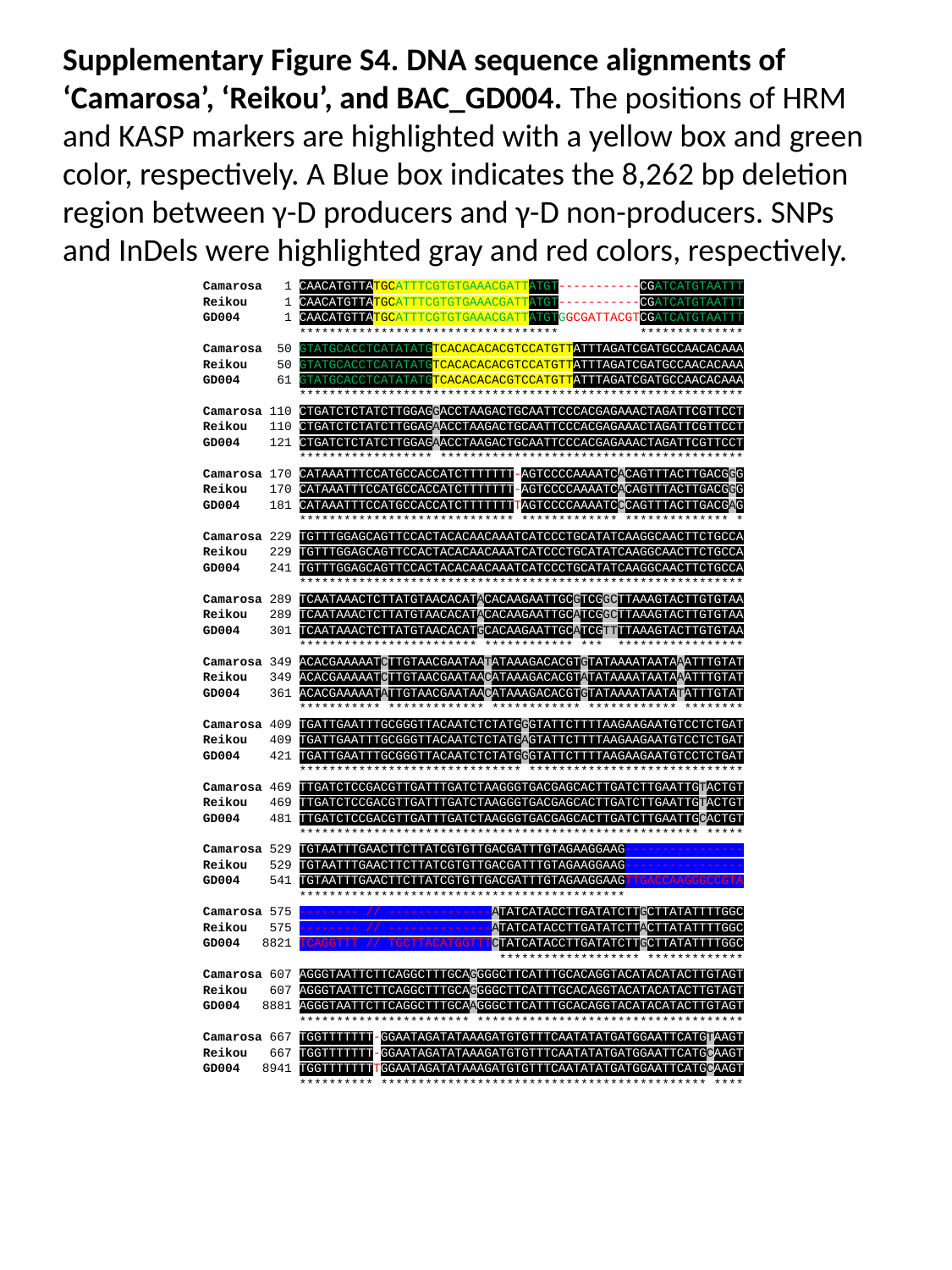

Supplementary Figure S4. DNA sequence alignments of ‘Camarosa’, ‘Reikou’, and BAC_GD004. The positions of HRM and KASP markers are highlighted with a yellow box and green color, respectively. A Blue box indicates the 8,262 bp deletion region between γ-D producers and γ-D non-producers. SNPs and InDels were highlighted gray and red colors, respectively.
Camarosa 1 CAACATGTTATGCATTTCGTGTGAAACGATTATGT-----------CGATCATGTAATTT
Reikou 1 CAACATGTTATGCATTTCGTGTGAAACGATTATGT-----------CGATCATGTAATTT
GD004 1 CAACATGTTATGCATTTCGTGTGAAACGATTATGTGGCGATTACGTCGATCATGTAATTT
        ***********************************           **************
Camarosa 50 GTATGCACCTCATATATGTCACACACACGTCCATGTTATTTAGATCGATGCCAACACAAA
Reikou 50 GTATGCACCTCATATATGTCACACACACGTCCATGTTATTTAGATCGATGCCAACACAAA
GD004 61 GTATGCACCTCATATATGTCACACACACGTCCATGTTATTTAGATCGATGCCAACACAAA
       ************************************************************
Camarosa 110 CTGATCTCTATCTTGGAGGACCTAAGACTGCAATTCCCACGAGAAACTAGATTCGTTCCT
Reikou 110 CTGATCTCTATCTTGGAGAACCTAAGACTGCAATTCCCACGAGAAACTAGATTCGTTCCT
GD004 121 CTGATCTCTATCTTGGAGAACCTAAGACTGCAATTCCCACGAGAAACTAGATTCGTTCCT
        ****************** *****************************************
Camarosa 170 CATAAATTTCCATGCCACCATCTTTTTTT-AGTCCCCAAAATCACAGTTTACTTGACGGG
Reikou 170 CATAAATTTCCATGCCACCATCTTTTTTT-AGTCCCCAAAATCACAGTTTACTTGACGGG
GD004 181 CATAAATTTCCATGCCACCATCTTTTTTTTAGTCCCCAAAATCCCAGTTTACTTGACGAG
   ***************************** ************* ************** *
Camarosa 229 TGTTTGGAGCAGTTCCACTACACAACAAATCATCCCTGCATATCAAGGCAACTTCTGCCA
Reikou 229 TGTTTGGAGCAGTTCCACTACACAACAAATCATCCCTGCATATCAAGGCAACTTCTGCCA
GD004 241 TGTTTGGAGCAGTTCCACTACACAACAAATCATCCCTGCATATCAAGGCAACTTCTGCCA
        ************************************************************
Camarosa 289 TCAATAAACTCTTATGTAACACATACACAAGAATTGCGTCGGCTTAAAGTACTTGTGTAA
Reikou 289 TCAATAAACTCTTATGTAACACATACACAAGAATTGCATCGGCTTAAAGTACTTGTGTAA
GD004 301 TCAATAAACTCTTATGTAACACATGCACAAGAATTGCATCGTTTTAAAGTACTTGTGTAA
        ************************ ************ ***  *****************
Camarosa 349 ACACGAAAAATCTTGTAACGAATAATATAAAGACACGTGTATAAAATAATAAATTTGTAT
Reikou 349 ACACGAAAAATCTTGTAACGAATAACATAAAGACACGTATATAAAATAATAAATTTGTAT
GD004 361 ACACGAAAAATATTGTAACGAATAACATAAAGACACGTGTATAAAATAATATATTTGTAT
        *********** ************* ************ ************ ********
Camarosa 409 TGATTGAATTTGCGGGTTACAATCTCTATGGGTATTCTTTTAAGAAGAATGTCCTCTGAT
Reikou 409 TGATTGAATTTGCGGGTTACAATCTCTATGAGTATTCTTTTAAGAAGAATGTCCTCTGAT
GD004 421 TGATTGAATTTGCGGGTTACAATCTCTATGGGTATTCTTTTAAGAAGAATGTCCTCTGAT
        ****************************** *****************************
Camarosa 469 TTGATCTCCGACGTTGATTTGATCTAAGGGTGACGAGCACTTGATCTTGAATTGTACTGT
Reikou 469 TTGATCTCCGACGTTGATTTGATCTAAGGGTGACGAGCACTTGATCTTGAATTGTACTGT
GD004 481 TTGATCTCCGACGTTGATTTGATCTAAGGGTGACGAGCACTTGATCTTGAATTGCACTGT
        ****************************************************** *****
Camarosa 529 TGTAATTTGAACTTCTTATCGTGTTGACGATTTGTAGAAGGAAG----------------
Reikou 529 TGTAATTTGAACTTCTTATCGTGTTGACGATTTGTAGAAGGAAG----------------
GD004 541 TGTAATTTGAACTTCTTATCGTGTTGACGATTTGTAGAAGGAAGTTGACCAAGGGCCGTA
        ********************************************
Camarosa 575 -------- // --------------ATATCATACCTTGATATCTTGCTTATATTTTGGC
Reikou 575 -------- // --------------ATATCATACCTTGATATCTTACTTATATTTTGGC
GD004 8821 TCAGGTTT // TGCTTACATGGTTTCTATCATACCTTGATATCTTGCTTATATTTTGGC
                                    ******************* *************
Camarosa 607 AGGGTAATTCTTCAGGCTTTGCAGGGGCTTCATTTGCACAGGTACATACATACTTGTAGT
Reikou 607 AGGGTAATTCTTCAGGCTTTGCAGGGGCTTCATTTGCACAGGTACATACATACTTGTAGT
GD004 8881 AGGGTAATTCTTCAGGCTTTGCAAGGGCTTCATTTGCACAGGTACATACATACTTGTAGT
        *********************** ************************************
Camarosa 667 TGGTTTTTTT-GGAATAGATATAAAGATGTGTTTCAATATATGATGGAATTCATGTAAGT
Reikou 667 TGGTTTTTTT-GGAATAGATATAAAGATGTGTTTCAATATATGATGGAATTCATGCAAGT
GD004 8941 TGGTTTTTTTTGGAATAGATATAAAGATGTGTTTCAATATATGATGGAATTCATGCAAGT
         ********** ******************************************** ****
