# Supplementary material for: Genomic Characterization of the Fruity Aroma Gene, FaFAD1, Reveals a Gene Dosage Effect on γ-Decalactone Production in Strawberry (Fragaria × ananassa)
Source: Front Plant Sci. 2021 May 4;12:639345. doi: 10.3389/fpls.2021.639345 (PMC8129584; doi:10.3389/fpls.2021.639345)
Supplement: Supplementary Table 1 — Bacterial Artificial Chromosome (BAC) libraries of three strawberry accessions. [file Data_Sheet_1.docx]

Supplementary Table S1. Bacterial Artificial Chromosome (BAC) libraries of three strawberry accessions.

|  | **Strawberry A:**  **FL11.77-96** | | **Strawberry B:**  **FL13.26-134** | | **Strawberry C:**  **FL14-101-225** | |
| --- | --- | --- | --- | --- | --- | --- |
| **Feature** | **γ-D producer** | | **γ-D producer** | | **γ-D Non-producer** | |
| **Enzyme** | AB1(BamH1) | AH3(HindIII) | BB1(BamH1) | BH3(HindIII) | CB1(BamH1) | CH3(HindIII) |
| **No. of Clone** | ~16,000 | ~16,000 | ~16,000 | ~16,000 | ~16,000 | ~16,000 |

| Supplementary Table S2. *De-novo* assembly results of six BAC clones. | | | |  |
| --- | --- | --- | --- | --- |
| **BAC clone** | **Feature** | **Count** | **Average length** | **Total bases** |
| BAC_GD001 | Total Reads | 7,608,116 | 149.14 | 1,134,677,463 |
|  | Matched reads | 6,379,719 | 149.33 | 952,711,185 |
|  | No. of Contigs | 3 |  | 110,483 |
|  | Reads in pairs | 6,185,450 |  |  |
| BAC_GD002 | Total Reads | 7,039,038 | 149.19 | 1,050,140,707 |
|  | Matched reads | 5,426,992 | 149.39 | 810,713,646 |
|  | No. of Contigs | 6 |  | 127,914 |
|  | Reads in pairs | 5,293,886 |  |  |
| BAC_GD003 | Total Reads | 11,435,790 | 149.04 | 1,704,402,075 |
|  | Matched reads | 8,912,377 | 149.26 | 1,330,298,770 |
|  | No. of Contigs | 9 |  | 178,457 |
|  | Reads in pairs | 8,505,162 |  |  |
| BAC_GD004 | Total Reads | 6,160,908 | 148.76 | 916,517,833 |
|  | Matched reads | 4,666,437 | 149.29 | 696,663,588 |
|  | No. of Contigs | 4 |  | 140,879 |
|  | Reads in pairs | 4,481,670 |  |  |
| BAC_GD005 | Total Reads | 7,084,286 | 149.2 | 1,057,007,170 |
|  | Matched reads | 6,468,005 | 149.41 | 966,405,205 |
|  | No. of Contigs | 7 |  | 122,783 |
|  | Reads in pairs | 6,190,052 |  |  |
| BAC_GD006 | Total Reads | 6,965,442 | 148.71 | 1,035,813,711 |
|  | Matched reads | 5,263,829 | 149.04 | 784,530,127 |
|  | No. of Contigs | 4 |  | 130,101 |
|  | Reads in pairs | 4,859,264 |  |  |

Supplementary table S3. Marker sequence information used in this study

| **Primer** | **Sequence** | **Tm**  **(**°C) | **Reference** |
| --- | --- | --- | --- |
| UFGDHRM5-F | CCTCGATCATAGCTACACTCTTTC | 62 | Noh et al. 2017 |
| UFGDHRM5-R | AGCCTTTGACGTGTCCTTATT |  |  |
| qFaFAD1-F | TCTGTACTCTACCGCCTTGC | 55 | Sánchez-Sevilla et al. 2014 |
| qFaFAD1-R | TCGTAGTGTGGCAGTGAAGG |  |  |
| LR_GD1-F | CACACACACGTCCATGTTATTT |  |  |
| LR_GD1-R | CCCTAGACTCATCACCATCATAC |  |  |
| NGD001-F | TGCATTTCGTGTGAAACGATTA | 62 |  |
| NGD001-R | AACATGGACGTGTGTGTGA |  |  |
| UFGDKASP-allele-1 | ATTTCGTGTGAAACGATTATGTG | 62 |  |
| UFGDKASP-allele-2 | TCGTGTGAAACGATTATGTC |  |  |
| UFGDKASP-common | CATATATGAGGTGCATACAAATTACATGAT |  |  |
| Allele-l Tail (FAM tail) | GAAGGTGACCAAGTTCATGCT |  |  |
| Allele-2 Tail (HEX tail) | GAAGGTCGGAGTCAACGGATT |  |  |
| GD_Rt1-F | GCCAACTCAAGAAAGCCATAC | 62 |  |
| GD_Rt1-R | TGGTAGAAGAGAGAGACCAAGA |  |  |
| FaGAPDH2-F | CCCAAGTAAGGATGCCCCCATGTTCG | 60 |  |
| FaGAPDH_R | TTGGCAAGGGGAGCAAGACAGTTGGTAG |  |  |
